# ALDH1L2 regulates reactive oxygen species and acinar-to-ductal metaplasia in the pancreas

**DOI:** 10.1038/s42255-026-01456-5

**Published:** 2026-04-01

**Authors:** Marc Hennequart, Loic Mervant, Julie Stockis, Jack Coomes, Martha M. Zarou, Louise Gerard, Virginie Tevel, Valérie Migeot, Steven E. Pilley, Younghwan Lee, Paul C. Driscoll, Nathalie M. Legrave, Christiaan F. Labuschagne, Fabio Zani, Alejandro Huerta Uribe, Rute Machado De Morais Ferreira, Eric C. Cheung, Ilaria Malanchi, James I. MacRae, Oliver D. K. Maddocks, Timotheus Y. F. Halim, Karen H. Vousden

**Affiliations:** 1 The Francis Crick Institute, London, UK; 2 NARILIS Institute, UNamur, Namur, Belgium; 3 University of Cambridge, CRUK Cambridge Institute, Cambridge, UK; 4 de Duve Institute, UCLouvain, Brussels, Belgium; 5 Department of Molecular Microbiology and Immunology, Keck School of Medicine, University of Southern California, Los Angeles, CA USA; 6 Luxembourg institute of Health, Luxembourg, Luxembourg; 7 Human Metabolomics, Faculty of Natural and Agricultural Sciences, North-West University Potchefstroom, Potchefstroom, South Africa; 8 Department of Pharmacology and Therapeutics, McGill University, Montreal, Quebec Canada; 9 School of Cancer Sciences, Wolfson Wohl Cancer Research Centre, University of Glasgow, Glasgow, UK

**Keywords:** Cancer metabolism, Pancreatic cancer, Metabolism

## Abstract

Acinar-to-ductal metaplasia (ADM) contributes to pancreatic repair after injury^1^. However, persistent ADM, combined with KRAS mutation, leads to the development of precancerous pancreatic intraepithelial neoplasia (PanIN) that can progress into pancreatic ductal adenocarcinoma (PDAC)^2^. While PDAC development is well documented, the metabolic rewiring that occurs during early events such as ADM is poorly understood. Here we show that aldehyde dehydrogenase 1 family member L2 (ALDH1L2), an NADPH-producing mitochondrial enzyme of the one-carbon pathway, limits reactive oxygen species (ROS) and formate production in pancreatic acinar cells. However, ALDH1L2 expression decreases progressively during ADM and is completely absent in pancreatic ductal cells. ALDH1L2 loss elevates ROS and promotes ADM in a model of pancreatitis and accelerates tumour progression in models of pancreatic cancer. We also show that formate increases during PDAC progression in mice and humans. Overall, our findings identify ROS as a driver of ADM and suggest that circulating formate may serve as a biomarker for PDAC progression.

## Abstract (web summary)

In the early stages of pancreatic ductal adenocarcinoma (PDAC) development, loss of ALDH1L2 expression elevates reactive oxygen species levels, driving accelerated acinar-to-ductal metaplasia. Decreased ALDH1L2 expression is accompanied by formate overflow, which can be used to monitor PDAC progression.

## Main

The pancreas regulates digestion and controls blood sugar. In the endocrine pancreas, the islets of Langerhans produce hormones such as insulin and glucagon that allow for the tight control of circulating glucose levels^3^. The exocrine pancreas, which represents up to 95% of the total pancreatic mass, is composed of acinar and ductal cells. The acinar cells, which constitute the majority of the exocrine pancreas, produce digestive enzymes such as proteases, lipase and amylase. Ductal cells produce an alkaline fluid that prevents aggregation of the digestive enzymes and water^3,4^.

Pancreatitis, which is characterized by damage and inflammation of the pancreas, can be caused by many factors, including alcohol consumption and gallstones. Acinar cells exhibit plasticity and in response to damage, they de-differentiate and take on some of the characteristics of ductal cells in a process termed acinar-to-ductal metaplasia (ADM)^5,6^. This is not a true transdifferentiation event as cells do not gain all the characteristic of ductal cells, rather adopting a progenitor-like phenotype^7^. They cease to secrete digestive enzymes and acquire an ability to proliferate that contributes to the regeneration of tissue after the damage is resolved. Furthermore, the ADM process can be reversed to re-establish normal pancreatic structure and function^1,7^. While acute pancreatitis can resolve over time, chronic pancreatitis is a known risk factor for pancreatic ductal adenocarcinoma (PDAC), the most common cancer of the pancreas^8,9^. Pancreatic cancer affected around half a million people worldwide in 2020 and has one of the lowest 5-year survival rates at around 10%. PDAC arises from a non-reversible ADM driven by activation of oncogenes such as *MYC* and *RAS*, which subsequently progresses into precancerous pancreatic intraepithelial neoplasia (PanIN)^2,10,11^. PanINs then accumulate other oncogenic mutations in genes such as *TP53*, *CDKN2A* and *SMAD4*, leading to the development of PDAC over time^2,8,12–14^.

The process of de-differentiation from acinar to ductal morphology can be induced by inflammation of the pancreas in response to damage, with a major role for cytokines secreted by pro-inflammatory macrophages. Several key transcription factors that control the expression of acinar and ductal genes are regulated during ADM, allowing for a switch of expression^7,15,16^. Most commonly, loss of expression of the acinar marker, amylase, and acquisition of expression of the ductal marker, CK19, is used to monitor ADM. However, a complex network of transcriptional changes accompanies pancreas regeneration, regulating signalling pathways that allow for both the activation and reversal of ADM^17,18^. Failure to properly control these pathways in a coordinated manner can result in an inability to resolve or repair the damage response, accompanied by an increase in malignant progression^19–22^. The rewiring of these pathways during ADM leads to changes in metabolism, yet the metabolic changes that occurs during this process is incompletely understood. Several studies have shown that increased levels of mitochondrial reactive oxygen species (ROS) play a role in promoting ADM^23,24^. However, it is also clear that excessive levels of ROS can be detrimental to the survival of acinar cells, leading to ferroptotic cell death^25^. This suggests that proper control of ROS-regulating systems is important in supporting the survival of acinar cells as they undergo ADM.

The mitochondrial enzyme aldehyde dehydrogenase 1 family member L2 (ALDH1L2) controls mitochondrial ROS by generating NADPH from one-carbon cycle intermediates, while also limiting the production of formate through the mitochondrial one-carbon pathway. Here, we show that ALDH1L2 is highly expressed in acinar cells and plays an important role in regulating ADM following pancreatic damage, with loss of ALDH1L2 resulting in accelerated ADM and enhanced tumorigenesis.

## Results

### ALDH1L2 expression in pancreatic acinar cells

**Fig. 1:**
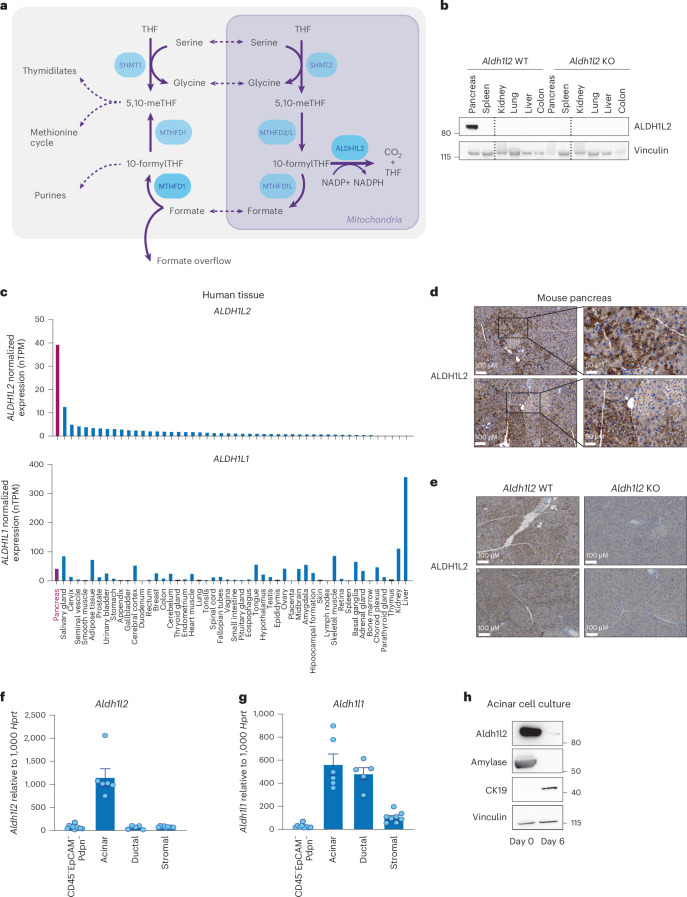
ALDH1L2 expression in normal pancreatic acinar cells. **a**, A scheme of cytosolic and mitochondrial one-carbon metabolism. Briefly, Serine hydroxymethyltransferase 1 (SHMT1) in the cytoplasm or SHMT2 in the mitochondria convert serine into glycine by donating a one-carbon unit to THF, producing 5,10-methylene-THF. Methylenetetrahydrofolate dehydrogenase enzymes (MTHFD2 and MTHFD2L in the mitochondria) oxidize 5,10-methylene-THF to 10-formyl-THF, regenerating NADPH. Mitochondrial 10-formyl-THF can then either be used to recycle THF by reducing NADP^+^ into NADPH by ALDH1L2 or can function as the substrate for the generation of formate by MTHFD1-like (MTHFD1L). Mitochondrial formate is transported to the cytoplasm and used to regenerate 10-formyl-THF and 5,10-methylene-THF by MTHFD1. Cytoplasmic 10-formyl-THF will be used for de novo purine synthesis whereas 5,10-methylene-THF will be used as a methyl donor in the methionine cycle or for thymidylate synthesis. Excess formate is excreted from the cell in a process termed formate overflow. **b**, ALDH1L2 expression determined by western blot in mouse tissues from *Aldh1l2* WT or KO mice and representative of both male and female mice and three independent experiments. **c**, *ALDH1L2* and *ALDH1L1* mRNA normalized expression (normalized transcripts per million (nTPM)) from human tissue samples extracted from the consensus dataset of the Human Protein Atlas^60^. **d**, ALDH1L2 staining on formalin-fixed paraffin-embedded (FFPE) sections from *Aldh1l2* WT mouse pancreas, representative of six individual mice. **e**, ALDH1L2 staining on FFPE sections from *Aldh1l2* WT and *Aldh1l2* KO mouse pancreas, representative of six individual mice. **f**,**g**, *Aldh1l2* (**f**) and *Aldh1l1* (**g**) mRNA expression measured by RT–qPCR in a subpopulation of healthy mouse pancreas isolated by flow cytometry. Acinar cells were gated as CD45^−^Epcam^+^CD133^+^SSC^hi^ gating, ductal cells as CD45^−^EpCAM^+^CD133^+^SSC^lo^ gating and stromal cells as CD45^−^EpCAM^−^Pdpn^+^. The remaining CD45^−^EpCAM^−^Pdpn^−^ cells contain mostly endothelial cells. Data represent the mean ± s.e.m. of individual female mice pooled from two independent experiments. CD45^−^EpCAM^−^Pdpn^−^ (*n* = 10), acinar (*n* = 6), ductal (*n* = 5) and stromal (*n* = 8). **h**, ALDH1L2, amylase and CK19 protein expression in ex vivo cultured acinar cells. The same samples were run on two separate blots and probed for ALDH1L2/CK19 and AMYLASE/VINCULIN. Representative of 2-4 independent experiments. Source data

**Extended Data Fig. 1:**
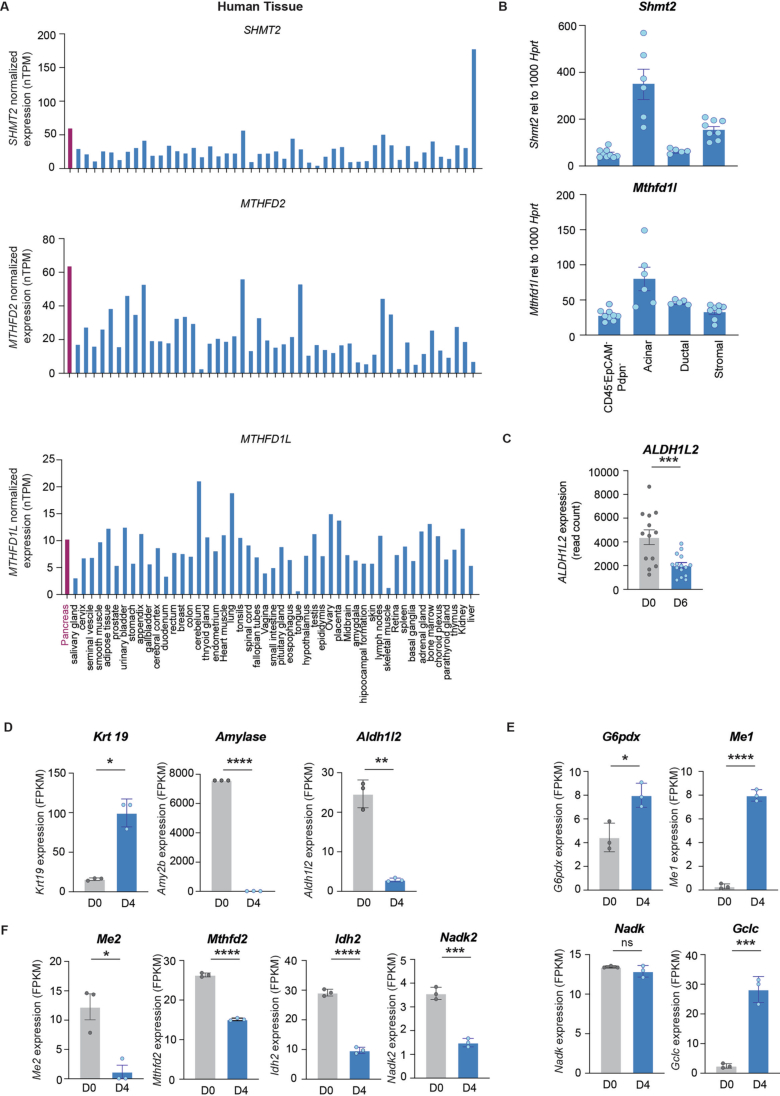
ALDH1L2 expression in normal pancreatic acinar cells. **A**) *SHMT2, MTHFD2* and *MTHFD1L* RNA normalized expression (nTPM) from human tissue samples extracted from the consensus dataset of the human protein atlas^60^. **B**). *Shmt2* and *Mthfd1l* mRNA expression measured by RT-qPCR in subpopulation of healthy mouse pancreas isolated by flow cytometry. Acinar cells were obtained by CD45^−^Epcam^+^CD133^+^SSC^hi^ gating, ductal cells by CD45^−^EpCAM^+^CD133^+^SSC^low^ gating, stromal cells as CD45^−^EpCAM^−^Pdpn^+^. Remaining non-immune cells are characterized as CD45^−^EpCAM^−^Pdpn^−^ endothelial cells. Data represented as the mean ± SEM of individual mice pooled from 2 independent experiments. CD45^−^EpCAM^−^Pdpn^−^ (n = 10), acinar (n = 6), ductal (n = 5), stromal (n = 8). **C**) mRNA expression of *ALDH1L2* in 3D *ex-vivo* cultures of human acinar cells at day 0 (n = 13) or at day 6 (n = 15) of ADM, extracted from available RNAseq data set (GSE179248). Data shown as mean ± SD. Unpaired t test with Welch correction, ***p-value = 0.001. **D**) mRNA expression of Cytokeratin 19 (*Krt19*), amylase (*amy2b*) and *Aldh1l2* expression in 2D *ex-vivo* cultures at day 0 (n = 3) and day 4 (n = 3) of mouse acinar cells extracted from available RNAseq data set (GSE240106). Data shown as mean± SD. Unpaired t test, ****p-value < 0.0001, **p-value = 0.0077, *p-value = 0.0138. **E**) mRNA expression of cytosolic enzymes (*G6pdx*, glucose-6-phosphate dehydrogenase; *Me1*, malic enzyme 1; *Nadk*, NAD^+^ Kinase; *Gclc*, glutamate cysteine ligase) in 2D *ex-vivo* cultures at day 0 (n = 3) and day 4 (n = 3) of mouse acinar cells extracted from available RNAseq data set (GSE240106). Data shown as mean ± SD. Unpaired t test, ****p-value < 0.0001, ***p-value = 0.006, *p-value = 0.0176. **F**) mRNA expression of mitochondrial enzymes (*Me2*, malic enzyme 2; *Mthfd2*, methylenetetrahydrofolate dehydrogenase 2; *Idh2*, isocitrate dehydrogenase 2; *Nadk2*, mitochondrial NAD^+^ kinase) in 2D *ex-vivo* cultures at day 0 (n = 3) and day 4 (n = 3) of mouse acinar cells extracted from available RNAseq data set (GSE240106). Data shown as mean ± SD. Unpaired t test, ****p-value < 0.0001, ***p-value = 0.003, *p-value = 0.0112. Source data

ALDH1L2 is an NADP^+^-dependent mitochondrial one-carbon cycle enzyme, responsible for the recycling of 10-formyl-tetrahydrofolate (THF) into THF and releasing CO_2_ (Fig. 1a). NADPH produced through this reaction has been shown to contribute to ROS regulation and antioxidant defence^26–30^. While ALDH1L2 expression has been detected in multiple types of cancer^26–28,30,31^, analysis of normal mouse tissue showed high levels of protein only in the pancreas, with very low levels of ALDH1L2 expression in other tissues (Fig. 1b). This pattern was also seen in humans, with high *ALDH1L2* RNA levels reported in the pancreas compared with most other normal tissues (Fig. 1c). This pancreas-restricted pattern of expression was not shared by the cytosolic *ALDH1L1* or the other mitochondrial one-carbon cycle enzymes *SHMT2*, *MTHFD2* and *MTHFD1L* (Fig. 1c and Extended Data Fig. 1a). Immunohistochemical analysis of mouse pancreas indicated that ALDH1L2 was strongly expressed in the exocrine pancreas compared with the endocrine pancreas (Fig. 1d), with loss of this staining in *Aldh1l2* knock-out mice (Fig. 1e). This led us to assess which cell population of the murine exocrine pancreas expresses *Aldh1l2*. To do so, we used specific markers to isolate acinar, ductal and stromal cells by flow cytometry^32^. Detection of *Aldh1l2* mRNA in these cell subsets showed that *Aldh1l2* expression was limited to acinar cells, with almost no expression in ductal or stromal cells (Fig. 1f). By contrast, the cytosolic enzyme *Aldh1l1* was expressed in all three populations (Fig. 1g). Similarly, other enzymes of the mitochondrial one-carbon metabolism, such as *Shmt2* and *Mthfd1l* were expressed in all major cell populations of the healthy pancreas (Extended Data Fig. 1b). This specific expression of *Aldh1l2* in acinar cells but not in ductal cells prompted us to query the fate of ALDH1L2 during ADM. ADM is a process that occurs during the initial response to pancreatic injury, whereby injured acinar cells undergo de-differentiation into ductal-like cells^7^. This process can be recapitulated ex vivo by placing pancreatic acinar cells into two-dimensional (2D) cell culture, which results, over time, in the loss of expression of the acinar marker amylase and in the increase in expression of the ductal marker, CK19 (ref.^33^) (Fig. 1h). In this system we also noted a loss of ALDH1L2 expression concomitant with the loss of the acinar marker, amylase (Fig. 1h). This loss was also observed at the RNA level in human acinar cells embedded in collagen and 2D cultured mouse acinar cells undergoing ADM^25,34^ (Extended Data Fig. 1c,d). Interestingly, expression of most mitochondrial enzymes implicated in NADPH production (*Me2*, *Mthfd2*, *Idh2* and *Nadk2*) is also downregulated during metaplasia while cytosolic enzymes (*G6pdx*, *Me1, Nadk* and *Gclc*) are upregulated (Extended Data Fig. 1e,f).

### A role for ALDH1L2 in response to pancreatic injury

**Fig. 2:**
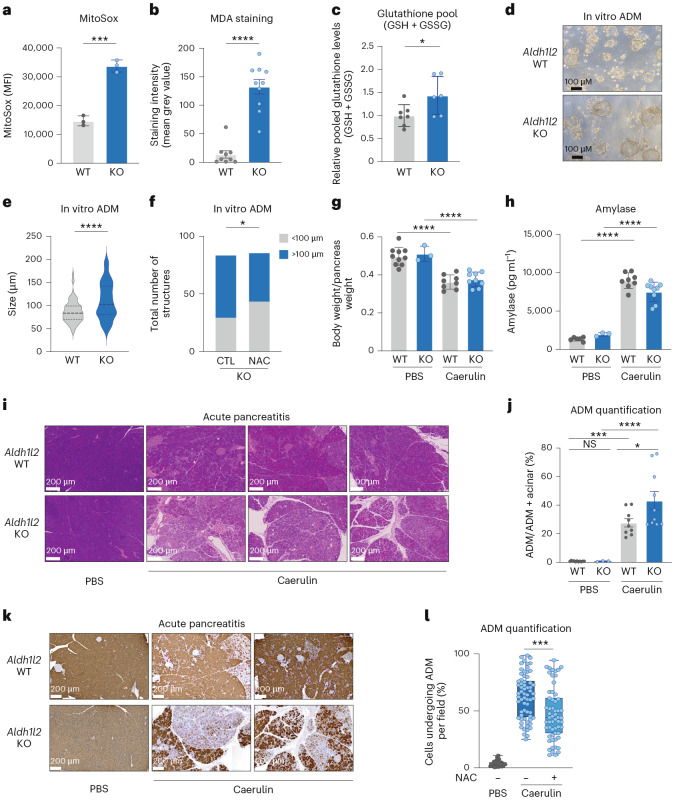
Deletion of *Aldh1l2* in models of ADM and acute pancreatitis. **a**, Mitochondrial ROS levels in *Aldh1l2* WT and KO ex vivo acinar cells, measured by flow cytometry. MFI, mean fluorescence intensity. Data represent the mean ± s.d. of triplicates. Unpaired *t*-test, ***P = 0.0003. **b**, Quantification of MDA immunostaining in *Aldh1l2* WT (*n* = 9) and KO (*n* = 10) mouse pancreas. Data represent the mean ± s.d. of the staining intensity (mean grey value). Unpaired Welch’s *t*-test, ****P < 0.0001. **c**, The relative levels of glutathione species (GSH + GSSG) in *Aldh1L2* WT (*n* = 7) or KO (*n* = 6) pancreas as measured by LC–MS. Data represent the mean ± s.d. of the relative peak area, representative of two independent experiments. Unpaired *t*-test, *P = 0.0373. **d**, Representative images of 3D acinar cell cultures undergoing ADM at day 3. **e**, The size of ductal structures measured at day 3 of ex vivo culture of WT or *Aldh1l2* KO acinar cells embedded in collagen. Data represent the mean ± s.d. of *n* = 139 WT and *n* = 93 KO ductal structures. Unpaired Welch’s *t*-test, ****P < 0.0001. **f**, The proportion of ductal structures bigger or smaller than 100 μM of *Aldh1l2* KO acinar cells after 3 days of culture with or without 300 μM NAC. CTL, control. Data represent a proportion of the total of *n* = 85 for untreated and *n* = 83 for NAC treated. Chi-square test, *P = 0.0430. **g**–**l**, For the induction of acute pancreatitis in WT or *Aldh1l2* KO mice, mice were injected intraperitoneally with either PBS or caerulein, representative of two independent experiments. The ratio of mouse body over pancreas weight at day 4. Data represent the mean ± s.d. of WT PBS (*n* = 10), *Aldh1l2* KO PBS (*n* = 3), WT caerulein (*n* = 8) or *Aldh1l2* KO caerulein (*n* = 9). One-way ANOVA with multiple comparisons, ****P < 0.0001 (**g**); the level of circulating amylase measured in the serum of mice. Data represent the mean ± s.d. of WT PBS (*n* = 10), *Aldh1l2* KO PBS (*n* = 3), WT caerulein (*n* = 8) and *Aldh1l2* KO caerulein (*n* = 9). One-way ANOVA with multiple comparisons, ****P < 0.0001 (**h**); representative images of H&E staining of mice with acute pancreatitis at day 4 (**i**); ADM quantification of H&E stained FFPE sections was performed with the HALO image analysis software. Data represent the mean ± s.e.m. of WT PBS (*n* = 10), *Aldh1l2* KO PBS (*n* = 3), WT caerulein (*n* = 9) and *Aldh1l2* KO caerulein (*n* = 10). One-way ANOVA with multiple comparison, ****P < 0.0001, ***P = 0.0002 and *P = 0.0432 (**j**); representative images of amylase (brown) and CK19 (pink) double staining of the pancreas of mice with acute pancreatitis at day 4 (**k**); the percentage of cells undergoing ADM as quantified by amylase and CK19 immunohistochemistry of *Aldh1l2* KO mice treated or not with 1% NAC in the drinking water. Data represent the mean ± s.d. (range (min–max): PBS (0.005–0.11), KO (0.25–0.99), KO-NAC (0.11–0.94); range percentile (25–75%): PBS (0.02–0.05), KO (0.44–0.77), KO-NAC (0.29–0.62)) of 28 fields of view obtained from *n* = 6 PBS-treated mice, 64 fields of view obtained from *n* = 10 caerulein-treated KO mice and 58 fields of view obtained from *n* = 10 caerulein and NAC-treated KO mice. One-way ANOVA, ***P = 0.0005 (**l**). Source data

**Extended Data Fig. 2:**
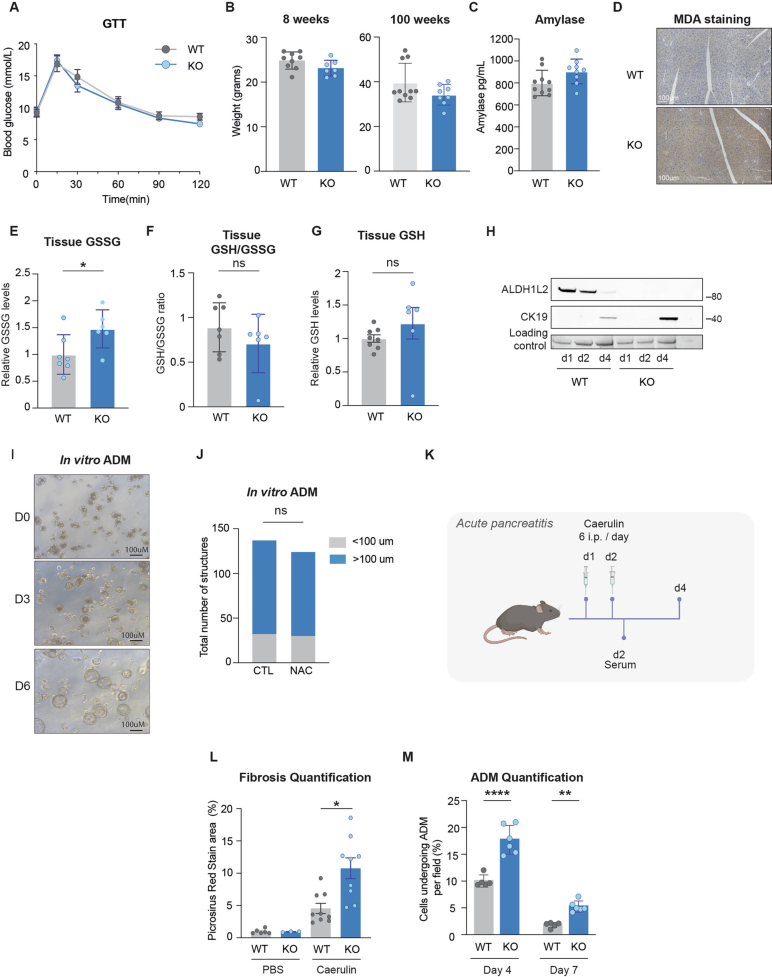
Deletion of *Aldh1l2* in models of acinar-to-ductal metaplasia and acute pancreatitis. **A**) Level of blood glucose in WT and *Aldh1l2* KO mice over time. **B**) Weight of WT and *Aldh1l2* KO mice at 8 weeks (WT, n = 9; KO, n = 7) and 100 weeks (WT, n = 9; KO, n = 7). Data represented as mean ± SD and representative of two independent experiments. Unpaired t-test. **C**) Basal level of serum amylase in WT (n = 10) and Aldh1l2 KO (n = 9) mice. Data represented as mean ± SD. Data representative of 2 independent experiments. Unpaired t test. **D**) Representative images of MDA staining quantified in Fig. 2b. E) Relative levels of oxidized glutathione (GSSG) in *Aldh1*L2 WT or KO pancreatic tissue as measured by LC-MS. Data represented as mean ± SD of the relative peak area of n = 7 mice for WT group and n = 6 for *Aldh1l2* KO group. Representative of 2 independent experiments. Unpaired t test, *p-value = 0.0376 **F**) Ratio of GSH to GSSG measured in *Aldh1l2* WT and KO pancreatic tissue. Data represented as mean ± SD of the relative peak area of n = 7 mice for WT group and n = 6 for *Aldh1l2* KO group. Representative of 2 independent experiments. Unpaired t test. **G**) Relative levels of reduced glutathione (GSH) in *Aldh1L2* WT or KO pancreatic tissue as measured by LC-MS. Data represented as mean ± SD of the relative peak area of n = 8 mice for WT group and n = 6 for *Aldh1l2* KO group. Representative of 2 independent experiments. Unpaired t test. **H**) ALDH1L2 and CK19 protein expression in *ex-vivo* culture of *Aldh1l2* WT and KO acinar cells undergoing ADM. Representative of 2 independent experiments. **I**) Representative images of 3D acinar cell cultures embedded in collagen undergoing ADM. **J**) Proportion of ductal structures bigger or smaller than 100 μm of *Aldh1l2* WT acinar cells after 4 days of culture with or without 300μM NAC. Data represented as a proportion of total of n = 139 for untreated and n = 126 for NAC-treated. Chi-square test. **K**) Scheme of acute pancreatitis protocol. WT or *Aldh1l2* KO mice received 6 intraperitoneal injections of either PBS or Caerulein, per day over two days. Serum for amylase quantification was collected at the end of the second day of injections. Mice were sacrificed at day 4 to assess acute pancreatitis severity. Created using BioRender **L**) Quantification of fibrosis by Picrosirius red staining of the pancreas of mice with acute pancreatitis at day 4. Data represented as mean ± SEM, Brown-Forsythe and Welch Anova test, * p-value = 0.0252. **M**) Quantification of ADM by immunohistochemistry at day 4 and day 7 of pancreatitis in WT (n = 5) and *Aldh1l2* KO (n = 6) mice. Data represented as mean ± SD. Two-way anova, ****p-value < 0.0001, **p-value = 0.0024. Source data

Despite the high level of ALDH1L2 expression in acinar cells, whole-body deletion of *Aldh1l2* did not lead to an obvious pancreatic phenotype in mice, with no significant differences in glucose homeostasis (Extended Data Fig. 2a), weight (Extended Data Fig. 2b) or basal pancreatic function (Extended Data Fig. 2c) compared with wild-type (WT) littermates. Consistent with a mitochondrial antioxidant role for ALDH1L2, we detected an increase in ROS in the *Aldh1l2* knockout (KO) acinar cells in vitro (Fig. 2a) and in vivo, as shown by quantification of malondialdehyde (MDA) staining in the pancreas (Fig. 2b and Extended Data Fig. 2d). This increase in ROS was accompanied by higher levels of glutathione in *Aldh1l2* KO pancreatic tissue, indicative of oxidative stress (Fig. 2c). In accordance with the role of ALDH1L2 in reducing NADP^+^ into NADPH, *Aldh1l2* KO pancreatic tissue exhibited a significant accumulation of oxidized glutathione (GSSG) (Extended Data Fig. 2e). However, reduced to oxidized glutathione ratios were not significantly decreased in pancreatic tissue lacking ALDH1L2 owing to a slight increase in reduced glutathione (GSH) levels (Extended Data Fig. 2f,g). This overall increase in GSH may reflect the induction of compensatory mechanisms deployed to deal with the increased levels of ROS. Numerous factors have been described to be changed during ADM, including ROS^23,24^. The association between ADM and increased ROS suggests that increased ROS following the downregulation or loss of ALDH1L2 may promote ADM. Indeed, we noted a stronger expression of CK19 in *Aldh1l2* KO acinar cells that underwent ex vivo ADM (Extended Data Fig. 2h). In an in vitro ADM assay, where mouse acinar cells embedded in a collagen matrix de-differentiate to form ductal structures (Extended Data Fig. 2i), we found that *Aldh1l2* KO acinar cells generated bigger ductal structures than control cells (Fig. 2d,e). Importantly, the addition of an antioxidant, *N*-acetyl-cysteine (NAC), led to a reduction in the proportion of bigger ductal structures in *Aldh1l2* KO but not WT cultures, supporting the suggestion that loss of ALDH1L2 drives ADM through increased ROS levels (Fig. 2f and Extended Data Fig. 2j).

In vivo, pancreatic injury in response to acute exposure to caerulein, a cholecystokinin analogue, results in ADM, followed by damage resolution and the re-acquisition of the normal acinar phenotype in a process spanning 7–10 days. Using a protocol to look at the early stages of this process (Extended Data Fig. 2k), we noted that loss of ALDH1L2 did not, at a macroscopic level, clearly exacerbate caerulein-induced pancreatitis, with similar increases in pancreas weight and circulating levels of amylase in control and *Aldh1l2* KO animals (Fig. 2g,h). However, in line with our in vitro data, *Aldh1l2* KO mice showed a stronger and more rapid transition to a ductal phenotype following exposure to caerulein (Fig. 2i–k), and the extent of ADM was limited by pretreatment of the mice with 1% NAC in the drinking water (Fig. 2l). These results are consistent with a role for ALDH1L2 in maintaining the acinar phenotype. Notably, we observed increased fibrosis in *Aldh1l2* KO mice at day 4 following caerulein treatment and a delay in the regeneration of acinar cells at day 7, consistent with a role for re-expression of ALDH1L2 in the recovery from pancreatic damage (Extended Data Fig. 2l,m).

### ALDH1L2 in the development of pancreatic cancer

**Fig. 3:**
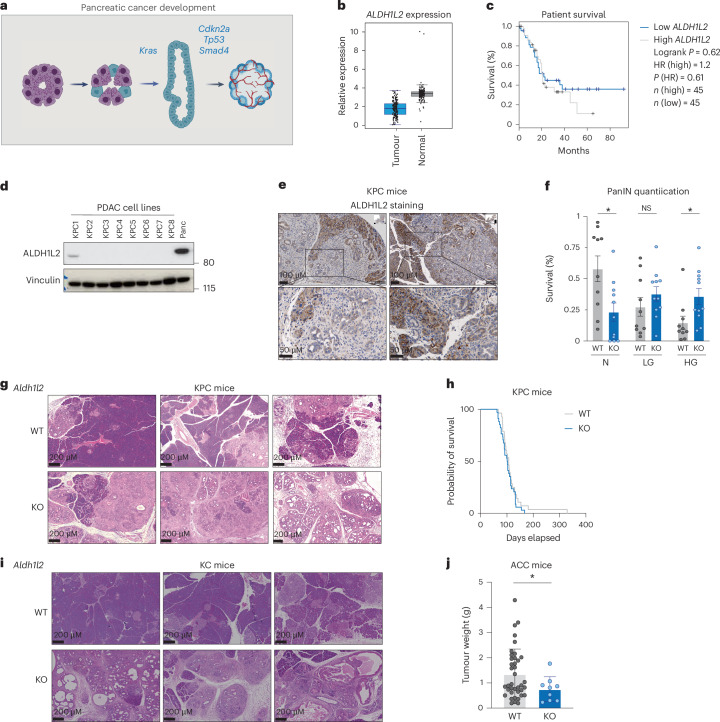
Deletion of *Aldh1l2* in mouse models of pancreatic cancer. **a**, A scheme of pancreatic cancer development. Briefly, acinar cells undergo ADM upon injury, acquiring a ductal phenotype that is irreversible when *Kras* mutation occurs. This leads to the formation of PanINs, which will further develop into PDAC upon accumulation of oncogenic drivers such as *Smad4*, *Tp53* and *Cdkn2a*. **b**, *ALDH1L2* mRNA expression in patients with pancreatic cancer from the PAAD TCGA dataset (*n* = 179) compared with normal tissue expression from GTEx data (*n* = 171). Data represent the mean ± s.d. and were obtained from GEPIA^61^. **c**, A survival curve of patients with *ALDH1L2*-high (*n* = 45) and *ALDH1L2*-low (*n* = 45) pancreatic cancer. The threshold for *ALDH1L2* expression classification was set as the median expression as measured by RNA sequencing and obtained from GEPIA^61^. **d**, ALDH1L2 protein expression in eight mouse PDAC cancer cell lines isolated from individual WT KPC mice, representative of three independent experiments. **e**, Representative immunohistochemistry staining of ALDH1L2 in the pancreas of 9-week-old KPC mice, representative of ten individual mice. **f**, Quantification of the area of normal tissue (N), low-grade PanINs (LG, PanIN 1 and 2) and high-grade PanINs (HG, PanIN 3 and PDAC) on H&E stained slides shown in **g**. Data represent the mean ± s.e.m. of a section at different levels from *Aldh1l2* WT (*n* = 7) or *Aldh1l2* KO (*n* = 6) mice. Two-way ANOVA with multiple comparison test, *P = 0.0421 for N and 0.0407 for HG. **g**, Representative images of H&E staining of the pancreas of 9-week-old *Aldh1l2* WT or KO KPC mice, representative of six individual mice for KO and seven for WT. **h**, Disease-free survival of WT (*n* = 35) and *Aldh1l2* KO (*n* = 29) KPC mice. **i**, Representative images of H&E staining of the pancreas of 15-week-old *Aldh1l2* WT or KO KC mice, representative of eight individual mice for both KO and WT. **j**, The weight of pancreatic acinar tumours from 16–18-week-old ELSV mice. Data represented the mean ± s.d. of *Aldh1l2* WT (*n* = 39) and *Aldh1l2* KO (*n* = 9). Welch’s *t*-test, *P = 0.0144. Panel **a** created with BioRender.com. Source data

**Extended Data Fig. 3:**
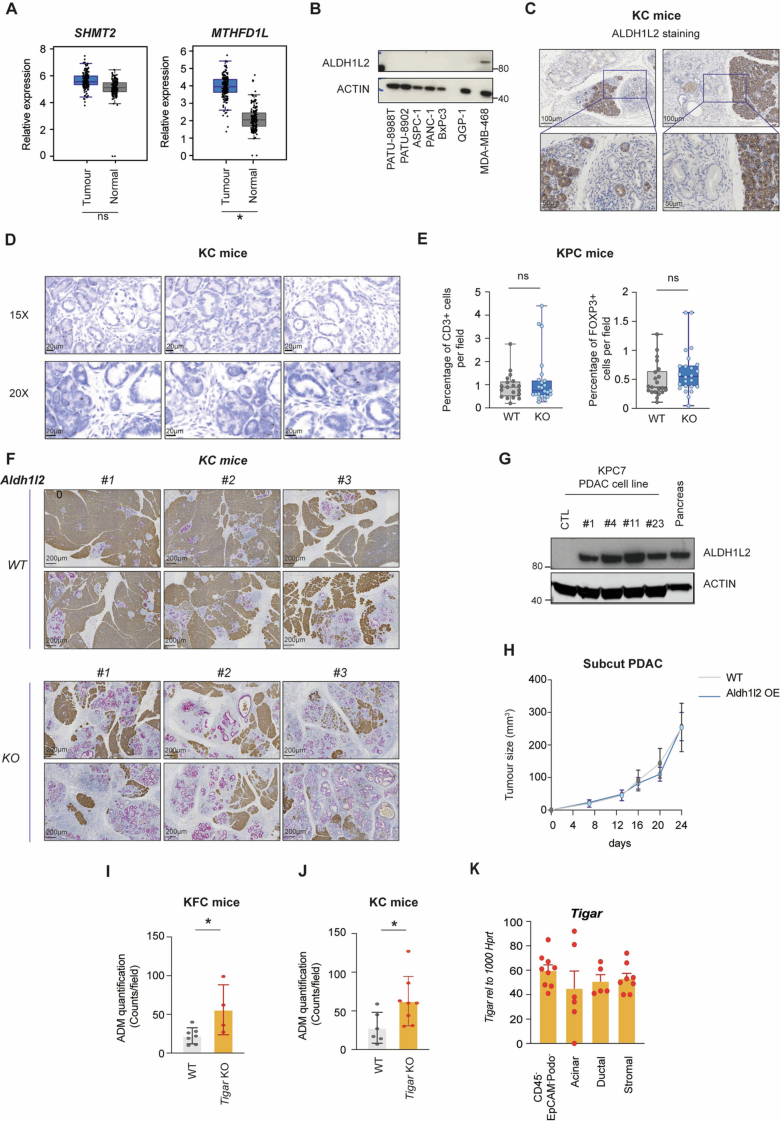
Deletion of *Aldh1l2* in mouse models of pancreatic cancer. **A**) *SHMT2 and MTHFD1L* mRNA expression in pancreatic cancer patients from the PAAD TCGA data set (n = 179) compared to normal tissue expression from GTEx data (n = 171). Data represented as mean ± SD and obtained from GEPIA^61^ **B**) ALDH1L2 protein expression in 6 human pancreatic cancer cell lines and one *ALDH1L2*-positive breast cancer cell line (MDA-MB-468). **C**) ALDH1L2 immunohistochemistry staining of pancreas tissue of 25-week-old KC mice. **D**) *Aldh1l2* mRNA staining by RNAscope of pancreas tissue from 15-week-old KC mice. **E**) Percentage of CD3^+^ (left panel) and FOXP3^+^ (right panel) cells in the pancreatic tumour tissue of 9 week old KPC mice as quantified by immunochemistry staining. Data represented as mean ± SD (range [min-max] for left panel: WT group [0.19-2.75] *Aldh1l2* KO group [0.26-4.39], right panel: WT group [0.11-1.28] KO group [0.05-1.66]) of n = 19 WT and n = 24 KO field of view taken on 7 mice from each background. Unpaired t test with welch correction for left panel and unpaired t test for right panel. **F**) Amylase (brown) and CK19 (pink) immunohistochemistry double staining in pancreatic tissue of 15-week-old WT and *Aldh1l2* KO mice. Representative of n = 10 per genotype. **G**) Ectopic over-expression of ALDH1L2 in clones following transfection of the pancreatic cancer cell line, KPC-7, as measured by western blot. **H**) Growth curve of pancreatic cancer cell line overexpressing ALDH1L2 (ALDH1L2-OE KPC-7, clone #23) or not after subcutaneous injection in syngeneic C57bl/6 mice. Data represented as ± SEM of n = 6 WT and n = 6 ALDH1L2 overexpressing tumours. Representative of 2 independent experiments. Two-way anova. **I**) ADM quantification in pancreatic tissue of 25-week-old WT (*Pdx1*^*Cre*^*;Kras*^*LSL-G12D/+*^;*Trp53*^*LSL-R172H/+*^;*Tigar*^*+/+*^, n = 6*)* and *Tigar* KO (n = 8) KC mice. Data is represented as mean ± SD. Unpaired t-test. *p-value = 0.0392. **J**) ADM quantification in pancreatic tissue of 6-week-old WT (*Pdx1*^*Cre*^*;Kras*^*G12D/+*^;*Trp53*^*fl/fl*^*;Tigar*^*+/+*^, n = 7*)* and *Tigar* KO (n = 4) KFC mice. Data is represented as mean ± SD. Unpaired t-test, *p-value = 0.0275. **K**) *Tigar* mRNA expression measured by RT-qPCR in subpopulation of healthy female mouse pancreas isolated by flow cytometry. Data represented as the mean ± SEM of individual mice pooled from 2 independent experiments. CD45^−^EpCAM^−^Pdpn^−^ (n = 10), acinar (n = 6), ductal (n = 5), stromal (n = 8). Source data

PDAC is characterized by the establishment of persistent ADM, which then evolves into PanIN before progressing into full malignancy (Fig. 3a)^35^. Consistent with the observation that ALDH1L2 expression is lost in ductal cells, we noted that in human PDAC, expression of *ALDH1L2* is lower in pancreatic cancer samples than in adjacent normal tissue, although levels of *ALDH1L2* expression did not correlate with patient survival (Fig. 3b,c). This is in contrast to other one-carbon cycle enzymes, such as *SHMT2* and *MTHFD1L*, the expression of which is unchanged or upregulated in PDAC compared with normal tissue (Extended Data Fig. 3a). Analysis of a panel of human PDAC cell lines revealed no detectable ALDH1L2 protein expression (Extended Data Fig. 3b). To assess the impact of ALDH1L2 loss in acinar cells on cancer development, we utilized well-established genetically engineered mouse models of pancreatic cancer that recapitulate, to some extent, human disease. In this model, *Ptf1a*^*Cre*^ is used to drive pancreatic expression of mutant KRAS (*Kras*^*LSL-G12D/+*^; KC) alone, with mutant p53 (*p53*^*LSL-R172H/+*^; KPC) or with loss of p53 (*p53*^*fl/+*^; KFC). As seen with the human cell lines, the majority of mouse PDAC cell lines derived from the KPC model were negative for ALDH1L2 expression (Fig. 3d). Furthermore, we noted an absence of ALDH1L2 staining in the tumour tissue of KPC mice with clear retention of ALDH1L2 protein in surrounding normal acinar cells (Fig. 3e). This loss of expression was confirmed in tumour tissue of KC mice (Extended Data Fig. 3c). Closer analysis at an early point in PDAC development of KC mice showed an almost complete loss of *Aldh1l2* RNA expression in the premalignant PanINs (Extended Data Fig. 3d). This suggests that loss of ALDH1L2 expression occurs early during malignant progression as cells acquire a ductal phenotype during ADM.

To test the importance of ALDH1L2 in the progression of PDAC, we crossed *Aldh1l2* KO mice into the KC and KPC models, which develop PanINs and subsequently PDAC with different latencies^36^. Analysis at early timepoints of the more aggressive KPC model revealed a greater proportion of higher-grade PanINs in the *Aldh1l2* KO mice, although this did not translate into a difference in survival (Fig. 3f–h). A recent study has shown that formate, a metabolite that is increased by loss of ALDH1L2, can enhance T cell activation and boost antitumour immunity^37^ but also favour T_reg_ differentiation^38^. While cells from the innate immune system rapidly infiltrate the pancreas upon acute damage, adaptive immune cells such as T cells infiltrate at a later stage of acute pancreatitis and during chronic inflammation such as the development of PanINs. While we did not observe significant differences in T cell or T_reg_ infiltration between WT or *Aldh1l2* KO tumours in KPC mice (Extended Data Fig. 3e), it remains possible that excreted formate could lead to a modulation of the immune response at early timepoints of the disease. Analysis of the pancreata of KC mice also showed a more rapid onset of PanINs in mice lacking ALDH1L2, with clear defects in pancreatic morphology within 15 weeks (Fig. 3i). This observation was validated with amylase/CK19 double staining (Extended Data Fig. 3f). Finally, we engineered an ALDH1L2 overexpressing KPC cell line to interrogate whether enforced expression of ALDH1L2 would limit the growth of cancer cells (Extended Data Fig. 3g), as previously observed in models of breast cancer^28^. Overexpression (OE) of ALDH1L2 did not impact the subcutaneous growth of these cells when injected in syngeneic C57Bl/6 mice, suggesting that loss of ALDH1L2 is a feature of ADM and not a selective advantage for established pancreatic cancer cells (Extended Data Fig. 3h).

To provide further support for the suggestion that increased ROS following loss of ALDH1L2 was responsible for the increase in ADM, we examined the impact of the loss of TIGAR—an unrelated protein with antioxidant functions^39^—in the PDAC models. These studies showed that *Tigar* KO KC and KFC mice also exhibited increased ADM (Extended Data Fig. 3i,j), consistent with a role for ROS control in driving ADM. Of note, in contrast to ALDH1L2, TIGAR is expressed throughout the stromal, acinar and ductal subpopulations in healthy pancreas (Extended Data Fig. 3k). Our previous work has shown that deletion of *Tigar* retards (rather than promotes) the development of PanINs^40^, indicating that the antioxidant function of TIGAR in ductal cells (where there is no expression of ALDH1L2) can help to support the survival of PanIN cells.

Finally, because of the high expression of ALDH1L2 seen in acinar cells, we used a mouse model of acinar cell carcinoma (ACC) driven by the SV40 T antigen (ELSV) to determine whether deletion of ALDH1L2 impacted the growth of these tumours^41^. ACC is an aggressive form of cancer that accounts for 1–2% of pancreatic neoplasms and up to 15% of paediatric cancer. It is driven by different oncogenes and is morphologically distinct from PDAC, consisting of hyperproliferative acinar cells^42^. ELSV mice that were WT or *Aldh1l2* KO were killed between 16 and 18 weeks to assess tumour growth and histopathology. Interestingly, *Aldh1l2* KO mice had significantly smaller tumours than their WT counterparts (Fig. 3j), indicating that maintenance of ALDH1L2 expression is required for the efficient development of acinar tumours. Taken together, these data support the proposal that loss of ALDH1L2 and increased ROS promote ADM and high-grade PanIN formation in the early stages of PDAC development. Conversely, in acinar tumours, loss of ALDH1L2 retards tumorigenesis.

### Regulation of formate production by ALDH1L2

**Fig. 4:**
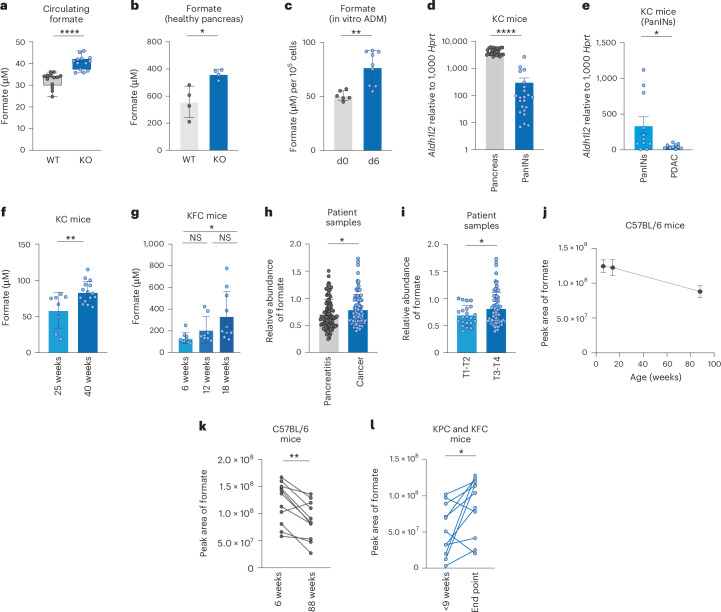
Circulating formate levels as a biomarker for pancreatic cancer detection. **a**, Quantification of formate by NMR in the blood of *Aldh1l2* WT (*n* = 13) or KO (*n* = 13) mice. Data represented the mean ± s.d. Unpaired *t*-test, ****P < 0.0001. **b**, Quantification of formate by NMR in the interstitial fluid of healthy pancreas from *Aldh1l2* WT (*n* = 4) or KO (*n* = 4) mice. Data represent the mean ± s.d. (range (min–max): WT (0.025–0.036), KO (0.036–0.046); range percentile (25–75%): WT (0.03–0.035), KO (0.037–0.042)). Unpaired *t*-test, *P = 0.0147. **c**, Quantification of formate in the supernatants of acinar cells undergoing ADM by NMR normalized per cell number. Data represent the mean ± s.d. of samples pooled from three independent experiments. Unpaired *t*-test, **P = 0.0015. **d**, mRNA expression of *Aldh1l2* measured by RT–qPCR of normal pancreas tissue (*n* = 26) or PanIN tissue (*n* = 22) from KC mice. Data represent the mean ± s.e.m. Unpaired *t*-test, ****P < 0.0001. **e**, Subclassification of the samples from tissues used in panel (**d**) by age of the KC mice, between 25 and 35 weeks old (PanIN; *n* = 11) and between 45 and 60 weeks old (PDAC; *n* = 10). Welsh *t*-test, *P = 0.0344. **f**, Quantification of formate by NMR in the plasma of 25-week-old (*n* = 8) and 40-week-old (*n* = 15) KC mice. Data represent the mean ± s.d. Unpaired *t*-test, **P = 0.0064. **g**, Quantification of formate by NMR in the plasma of 6-week-old (*n* = 9), 12-week-old (*n* = 8) and 18-week-old (*n* = 10) KFC mice. One-way ANOVA with multiple comparisons, *P = 0.0248. **h**, Normalized relative abundance of formate in the plasma of patients with pancreatitis (*n* = 97) and pancreatic cancer (*n* = 98), measured by LC–MS. Data represent the mean ± s.d. Unpaired *t*-test, *P = 0.0499. **i**, Normalized relative abundance of formate in the plasma of patients with pancreatic cancer stratified by tumour size, T1–T2 (*n* = 23) and T3–T4 (*n* = 75), measured by LC–MS. Data represent the mean ± s.d. Welch’s *t*-test, *P = 0.0289. **j**, Formate levels measured by LC–MS in the plasma of healthy C57BL/6 mice over time. Data represent the mean ± s.d. of *n* = 11 mice sequentially sampled at 6, 14 and 88 weeks of age. **k**, Formate levels, measured by LC–MS, in the plasma of healthy C57Bl/6 mice sampled at 6 weeks (*n* = 11) and 88 weeks (*n* = 11). Paired *t*-test, **P = 0.0014. **l**, Levels of formate in plasma of KPC and KFC mice (*n* = 11) sampled before 9 weeks of age and at a humane end point measured by LC–MS. Paired *t*-test, *P* = 0.0272. Source data

**Extended Data Fig. 4:**
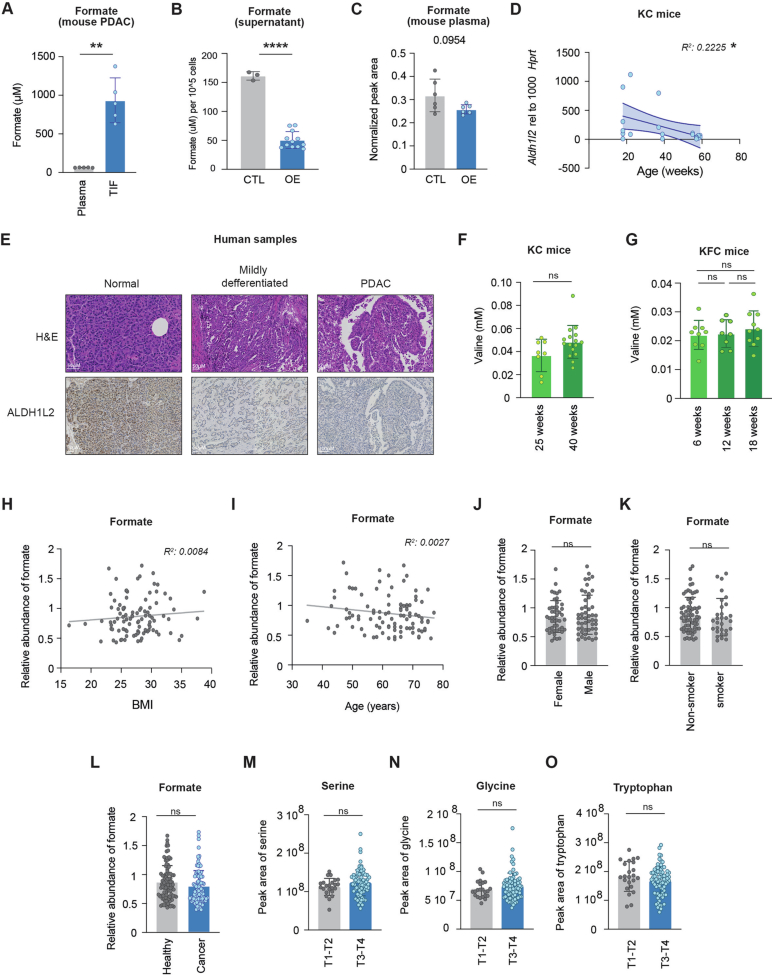
Circulating formate levels as biomarkers for pancreatic cancer detection. **A**) Quantification of formate in the interstitial fluid (TIF) and plasma by NMR of pancreatic tumours grown subcutaneously in C57bl/6 mice (n = 5). Data represented as mean ± SD, unpaired Welch’s t-test, **p-value = 0.0026. Representative of two independent experiments. **B**) Quantification of formate by NMR in the supernatants of mouse pancreatic cancer cell line, KPC-7 (CTL) or KPC-7 clones shown in extended data 3G, over-expressing ALDH1L2 (OE). Data represented as mean ± SD of n = 3 replicates for control and n = 12 replicates for OE cells (pool of 3 replicates per clone). Unpaired t test, ****p-value < 0.0001. **C**) Normalized peak area of formate as measured by LC-MS in the blood of C57bl/6 mice bearing KPC-7 (CTL) or ALDH1L2 overexpressing (OE) subcutaneous tumours. Data represented as mean ± SD of n = 6 mice per group. Unpaired Welch’s t test, p-value = 0.0954. **D**)Linear regression of the mRNA expression of *Aldh1l2* in PanIN tissue of KC mice. Samples from Fig. 4d. *p-value = 0.0309. **E**) H&E and ALDH1L2 immunohistochemistry staining of tissue from human normal pancreas, mildly differentiated pancreas and pancreatic adenocarcinoma. **F**) Levels of valine measured by NMR in the plasma of 25 week (n = 8) and 40 week (n = 16) old KC mice. Data represented as mean ± SD, unpaired t-test. **G**) Levels of valine measured by NMR in the plasma of 6 week (n = 9), 12 week (n = 8) and 18 week (n = 10) old KFC mice. Data represented as mean ± SD, One-way anova with multiple comparison. **H**) Linear regression of the normalized relative abundance of formate, as measured by LC-MS, in healthy individuals related to BMI (n = 97). **I**) Linear regression of the normalized relative abundance of formate, as measured by LC-MS, in healthy individuals related to age (n = 97). **J**) Normalized relative abundance of formate in male (n = 98) and female (n = 99) healthy individuals. Data represented as mean ± SD. Unpaired t-test. **K**) Normalized relative abundance of formate in healthy non-smokers (n = 67) and smokers (n = 30). Data represented as mean ± SD. Unpaired t-test. **L**) Normalized relative abundance of formate in healthy individuals (n = 96) and patients with pancreatic cancer (n = 96). Data represented as mean ± SD. Unpaired t-test. **M-O**) Peak area of serine (**M**), glycine (**N**) and tryptophan (**O**) in patients with T1-T2 tumours (n = 23) and patients with T3-T4 tumours (n = 75). Data represented as mean ± SD. Unpaired t-test. Source data

While ALDH1L2 has been most commonly associated with antioxidant defence, we showed recently that it can also regulate the production of formate, with loss of ALDH1L2 resulting in increased formate production in cancer cells^28^. In this study, we confirmed that *Aldh1l2* KO mice have higher levels of formate in the blood and in the interstitial fluid (TIF) of normal pancreas (Fig. 4a,b). Accordingly, ex vivo cultures of acinar cells undergoing ADM exhibited higher concentration of formate in their supernatant, concomitant with a loss of ALDH1L2 expression during this process (Figs. 1h and 4c). Formate is a building block for purine synthesis but is rapidly excreted if produced in excess, leading to formate overflow^43^. Previous studies have shown that mouse models of breast and colon cancer exhibit increased levels of circulating formate compared with tumour-free mice^44^. Interestingly, concentrations of formate in the TIF of ALDH1L2-negative PDAC cell lines grown subcutaneously in mice were up to 20-fold higher compared with the circulating levels (Extended Data Fig. 4a). This high concentration of formate (~1 mM) could be due to the absence of ALDH1L2 in the PDAC cells. Conversely, OE of ALDH1L2 in KPC cells led to the reduction of formate overflow, both in vitro and in vivo, as shown by reduced formate levels in the supernatant of ALDH1L2 overexpressing cells and plasma of ALDH1L2-OE tumour-bearing mice (Extended Data Fig. 4b,c). These data suggested that the progressive loss of ALDH1L2 expression that occurs upon the de-differentiation of acinar cells into ductal PanINs could lead to increased circulating formate that may serve as an early biomarker for PDAC detection.

The dismal prognosis faced by patients with pancreatic cancer is partly owing to the late presentation of symptoms, leading to over 50% of patients being diagnosed with advanced cancers^45^. We set out to test whether an increase in circulating levels of formate, driven by a loss of ALDH1L2 expression, can help detect pancreatic cancer. As expected, we observed a reduction in *Aldh1l2* expression in PanIN samples compared with normal pancreas of KC mice (Fig. 4d) and a further decrease in mice carrying PDAC compared with PanIN (Fig. 4e). In this relatively slowly developing PDAC model, a progressive loss of *Aldh1l2* expression in the pancreas was observed over time (Extended Data Fig. 4d). A similar decline of ALDH1L2 expression was seen during progression from normal human pancreas, through mildly differentiated PanIN lesions to full PDAC (Extended Data Fig. 4e). These observations prompted us to explore the possibility that changes in formate levels accompany early stages of PDAC development. We detected a clear increase in circulating formate levels in 40-week-old KC mice, compared with 25-week-old counterparts that retained higher levels of pancreatic *Aldh1l2* expression (Fig. 4f). Importantly, in this KRAS-only driven model, progression to PDAC occurs around 48 weeks. A similar increase in circulating formate levels was measured between 6 and 18 weeks in the more aggressive KFC model, where PDAC starts to develop at around 6 weeks (Fig. 4g). In both cases, the change in formate levels was detected at early timepoints of the disease. In this model, we were not able to detect changes in plasma valine, a branched-chain amino acid that is associated with an increased risk of PDAC diagnosis^46^ (Extended Data Fig. 4f,g). Finally, we queried circulating formate levels in healthy individuals and patients suffering from either pancreatitis or pancreatic cancer. In this cohort, circulating formate levels were not correlated to smoking, sex, BMI or age in healthy participants (Extended Data Fig. 4h–k). Although circulating formate levels did not discriminate between healthy and all cancer patients (Extended Data Fig. 4l), we observed a significant increase of formate in the plasma of patients suffering from pancreatic cancer compared with patients with pancreatitis (Fig. 4h). Furthermore, patients carrying larger tumours (T3 and T4 according to TMN classification) exhibited significantly higher circulating formate levels (Fig. 4i). This increase was specific to formate and was not seen for metabolites that can contribute to formate production such as serine, glycine and tryptophan (Extended Data Fig. 4m–o). Taken together, the mouse and human data suggest that circulating levels of formate driven by loss of ALDH1L2 correlate with increased tumour burden. Tracking formate levels in the circulation over time in individual mice showed a slight decrease in healthy mice, a trend that was also observed in the healthy patient data (Fig. 4j,k and Extended Data Fig. 4i). However, in ten mice bearing pancreatic tumours,we observed a significant increase in circulating formate in samples obtained when the mice had reached humane end point compared with samples taken early in the development of their disease (Fig. 4l). Taken together, these data highlight the potential use of circulating formate detection for monitoring pancreatic cancer progression.

## Discussion

Pancreatic ADM has been well described at the transcriptional level^47^ and previous work has suggested that ROS levels can determine the fate of acinar cells. Exposure of acinar cells to peroxide to increase ROS levels accelerates ex vivo ADM^23^ and treatment with antioxidants limits ex vivo ADM and tumour development^24,48,49^. However, ROS can play different roles in promoting or limiting different stages of tumorigenesis in the pancreas and a dynamic regulation of ROS levels appears to be required for full malignant progression^40,49^.

In this study, we show that ALDH1L2—an NADPH-producing mitochondrial enzyme—is exclusively expressed in the acinar cells of the pancreas and controls ROS levels in these cells. Loss of ALDH1L2 promotes ADM both in models of ex vivo ADM and in caerulein-induced pancreatitis. Furthermore, loss of ALDH1L2 in the pancreas led to faster progressing pancreatic cancer in *Kras*^*G12D*^-only driven KC and *Kras*^*G12D*^ *Tp53*^*R172H*^-driven KPC mouse models. As ALDH1L2 is not expressed in ductal cells, these results indicate that increased ROS in acinar cells leads to a more rapid development of ADM, promoting an increase in premalignant PanINs and PDAC, despite the loss of ALDH1L2 expression in these lesions.

The importance of ROS control in ADM was confirmed in *Tigar* KO mice. Loss of TIGAR also increases ROS and—as seen in the *Aldh1l2*-deficient mice—this is accompanied by an increased ADM. However, loss of TIGAR decreases the appearance of PanINs^40^, probably reflecting a role for TIGAR-mediated antioxidant defence in ductal cells, where TIGAR but not ALDH1L2 is expressed. Furthermore, the accompanying paper shows that deletion of glucose-6-phosphate dehydrogenase (G6PD) and malic enzyme (ME1), both of which drives NADPH production and antioxidant defence, also increases ROS-dependent ADM (Radyk et al., manuscript in press).Taken together, these results underline the importance of controlling ROS levels in acinar cells of the pancreas. It is interesting to note that the expression of mitochondrial NADPH-producing enzymes (including ALDH1L2) decreases during ADM, while the expression of cytosolic enzymes (such as G6PD) increases (Extended Data Fig. 1e,f). These observations could suggest that in response to damage, an increase in mitochondrial ROS drives ADM, while enhanced cytosolic antioxidant defence prevents excessive ROS accumulation. As ALDH1L2 is part of the mitochondrial one-carbon pathway, the results imply a critical role of serine metabolism in ADM. Interestingly, a recent study has shown a requirement for de novo serine and glutathione synthesis for ADM development^50^, further supporting the suggestion that excessive ROS may be detrimental to this process and underscoring the importance of the ROS balance in acinar cells.

While acinar cells of the pancreas express by far the highest levels of ALDH1L2, other cell types show lower levels of expression, including several components of the immune system. Publicly available transcriptomic data show that *Aldh1l2* is expressed in circulating granulocytes and non-classical monocytes^51^. These immune populations are important during tissue repair and regeneration following injury^52,53^. Our ex vivo acinar cell experiments show the cell-intrinsic effect of loss of ALDH1L2 in acinar cells. However, since our mouse model carries a whole-body deletion of *Aldh1l2*, it is possible that the overall in vivo effect reflects changes in other cell populations that play a role in regulating the response to damage and oncogene activation. Further analysis of tissue-specific ALDH1L2 deletion will clarify this point.

In addition to controlling NADPH production, ALDH1L2 also functions to limit formate synthesis. The loss of ALDH1L2 expression as cells acquire a more ductal phenotype is accompanied by an increase in formate overflow. We note recent studies showing that excess formate can support other cells in the tumour microenvironment^37,54^. Formate increases T cell activity, suggesting that the presence of this metabolite would favour an immune response to damage or tumour formation^37^. However, we did not detect changes in T cell numbers in PDAC arising in *Aldh1l2* null mice compared with WT mice, although it remains possible that the loss of ALDH1L2 expression and increase in formate support immune cell activity at different stages of tumorigenesis. It would also be of interest to determine whether ALDH1L2 expression or formate levels correlate with the response to immunotherapies in PDAC and other tumours.

While the normal pancreas is composed predominantly of ALDH1L2-expressing acinar cells, the gradual replacement of normal tissue with ALDH1L2-negative tumour cells results in an increase in formate overflow that is clearly detected in the PDAC tumour interstitial fluid. We noted that formate levels were correlated with disease progression in KC and KFC mouse models, although this was not seen previously in the KPC model^44^. Both studies show a high degree of variability in formate levels between mice, prompting us to monitor formate in individual mice over time. Interestingly, individual tumour-bearing mice exhibited an increase in circulating formate with disease progression while healthy mice did not show such an increase over time. In human samples, circulating formate levels were higher in patients bearing larger tumours. Therefore, while circulating formate levels were unable to discriminate between healthy participants and patients with pancreatic cancer, our data suggest that tracking formate levels over time in individuals could yield a biomarker for pancreatic cancer progression.

## Methods

### Mouse experiments

All experiments were conducted in compliance with the UK Home Office-approved project licences and personal licences (Animals Scientific Procedures Act 1986) and within institutional welfare guidelines of the Francis Crick Institute (reviewed and approved by the Francis Crick Animal Welfare Ethical Review Body), the CRUK Scotland Institute (reviewed and approved by the University of Glasgow and UK Home Office) and the CRUK Cambridge Institute (with approval from CRUK-CI Animal Welfare Ethical Review Body). In accordance with these project licences, tumour size never exceeded 1.2 cm in diameter. Mice (three to five per cage, individually ventilated cages, Tecniplast) had ad libitum access to food (2018 Teklad global diet (Envigo) autoclaved before use) and water and were kept in a 12 h day/night cycle (7:00 to 19:00) in rooms at 22 °C and 55% humidity. Mice were acclimatized to their environment for at least 1 week before experimentation. Both male and female mice between 8 and 12 weeks of age (Extended Data Table 1) were used in the experiments and were randomly assigned to experimental groups and data were collected in a blinded manner. C57BL/6 were obtained from the in-house breeding facilities. *Tigar* KO, *Tigar* KO pancreatic cancer models and *Aldh1l2* KO mice were obtained as previously described^28,40^. For Fig. 4d,e and Extended Data Fig. 4d, *Ptf1a*^*Cre/+*^ *Kras* ^*LSL-G12D*^ mice (KC mice) were provided by Prof. Brindle (CRUK-CI). For Figs. 3 and 4 and Extended Data Figs. 3 and 4, unless indicated otherwise, *Trp53*^*+/LSL-R172H*^, *Kras*^*+/LSL-G12D*^, *Trp53*^*+/fl*^, *Ptf1a*^*Cre*^ strains were crossed to obtain KFC (*Ptf1a*^*Cre*^; *Kras*^*+/LSL-G12D*^; *T rp53*^*+/fl*^) and KPC (*Ptf1a*^*Cre*^, *Kras*^*+/LSL-G12D*^, *Trp53*^*+/LSL-R172H*^) mice^55,56^. For *Aldh1l2* knock outs, the *Aldh1l2* KO strain was used to breed into the KFC to obtain KPC and KFC mice bearing *Aldh1l2* deletion in C57Bl/6 background. For Fig. 3j, the acinar cancer model, B6.Cg-Tg(Ela1-TAg*)289Mjt/J was obtained from the Jackson laboratory (no. 008247) and crossed with the *Aldh1l2* KO strain. Mice were kept to a maximum of 18 weeks of age.

For acute pancreatitis experiments, mice received an injection each hour over 6 h of 75 μg kg^−1^ Caerulein (Sigma) or endotoxin-free PBS intraperitoneally per day for 2 days. At the end of day 2, blood was collected from the saphenous vein. For NAC treatment, mice were given a 1% NAC solution in the drinking water ad libitum 1 week before pancreatitis onset. Mice were then killed either on day 4 or day 7.

For the glucose tolerance test (Extended Data Fig. 2a), mice were moved to a fresh cage and fasted for 6 h. Mice were then administered an oral gavage of 2 mg kg^−1^ of glucose (Merck, 158968) at 30 s/1 min intervals. Blood glucose was measured using a glucometer (Accu-CHEK) at the following timepoints: 0 (before injection), 15, 30, 60 and 120 min. Blood glucose was obtained by cutting the tip of the tail and collecting about 5 μl of blood.

For Extended Data Figs. 3h and 4a,c, 5 × 10^5^ cells from a KPC PDAC cell line were injected subcutaneously in syngeneic C57BL/6 mice. Tumours were collected after 15 days of growth to obtain TIF.

### Cell culture

For Extended Data Fig. 3b, PATU-8988T, BxPc3, PANC-1 and ASPC-1 PATU-8902 pancreatic cancer cell lines and MDA-MB-468 breast cancer cell line were obtained from the Francis Crick Institute Cell Services Science Technology Platform. The QGP-1 pancreatic cancer cell line was purchased from Generon and deposited at the Francis Crick Institute Cell Services, which ensures various quality controls such as mycoplasma testing, STR profiling and species validation of all cell lines. All cell lines were kept in DMEM (Thermo Fisher) supplemented with 10% fetal bovine serum (FBS) and penicillin–streptomycin at 37 °C in a humidified atmosphere of 5% CO_2_.

Mouse pancreatic cancer cell lines were obtained from *Ptf1a*^*Cre+*^, *Kras*^*+/LSL-G12D*^, *Trp53*^*+/LSL-R172H*^ mouse tumours collected in PBS with 1% penicillin–streptomycin and then finely minced. Minced tissues were then incubated with collagenase type 1 (200 U ml^−1^, Gibco) and dispase (2.4 U ml^−1^, Gibco) in HBSS for 1 h at 37 °C for cell dissociation. After washing 2× in HBSS, cell pellets were resuspended and grown in DMEM containing 10% FBS, 2 mM l-glutamine and 1% penicillin–streptomycin.

For stable OE of ALDH1L2 in the KPC-7 cell line. HEK293T cells were transfected with lentiviral plasmids containing the *Aldh1l2* ORF pLV[Exp]-Puro-CMV>mAldh1l2:P2A:EGFP (VB210527-1256nfw) together with psPAX2 (Addgene, 12260) and VSV.G (Addgene, 14888) using jet-PRIME reagent (Polyplus transfection). After 24 h incubation, medium was changed and 48 h later, the viral particle containing-medium was filtered (0.45 mm) and mixed with polybrene (4 mg ml^−1^, Sigma-Aldrich). The medium containing lentiviruses was incubated with the target cells for 24 h. Cells were then selected for 3 weeks in puromycin. OE-ALDH1L2 and equivalent empty vector cells were single-cell cloned in 96-well plate and selected on the basis of GFP expression by flow cytometry

For ex vivo 2D cultures of acinar cells, pancreata were collected from *Aldh1l2* KO or WT mice and immediately put in ice-cold, calcium-free, HBSS. Tissues were washed with calcium-free HBSS, then digested in 200 U ml^−1^ collagenase type 1 (Gibco) solution containing trypsin inhibitor 0.25 mg ml^−1^ (Sigma) and 10 mM HEPES (Lonza). The cell suspension was washed three times using HBSS containing 10 mM HEPES and 2.5% FBS. Finally, the cells were resuspended in Waymouth medium (containing 2.5% FBS, 1% penicillin–streptomycin, 0.25 mg ml^−1^ trypsin inhibitor and 25 ng ml^−1^ EGF (Preprotech)) and filtered through a 100 µm cell strainer then plated in 6-well plate. The next day, the cell suspension was transferred to a collagen-coated 6-well plate with 50 µg ml^−1^ PureCol solution (Sigma). Media were replenished every 2 days to avoid loss due to evaporation. On the day of collection, cells were scraped, washed with PBS and stored at −80 °C until further analysis.

For ex vivo 3D cultures of acinar cells^57^, 12-well plates were precoated with 240 μl of Matrigel Membrane Matrix Growth factor reduced (Corning). Pancreata from mice were collected and rinsed twice in 5 ml ice-cold calcium-free HBSS (Gibco). Tissues were minced then centrifuged for 2 mins at 450*g* and 4 °C. HBSS was discarded and tissues were digested using 200 U ml^−1^ collagenase type 1 (Gibco) solution containing trypsin inhibitor 0.25 mg ml^−1^ (Sigma) and 10 mM HEPES (Lonza) for 15–20 min at 37 °C. Digestion was stopped by adding 10 ml of ice-cold HBSS with 2.5% FBS. Cells were washed three times using HBSS containing 10 mM HEPES and 2.5% FBS, then resuspended in HBSS with 30% FBS and centrifuged at 233*g* for 2 min. Following resuspension in 7 ml Waymouth medium (containing 2.5% FBS, 1% penicillin–streptomycin, 0.25 mg ml^−1^ trypsin inhibitor and 25 ng ml^−1^ EGF (Preprotech)), the cells were filtered through a 100 μm cell strainer. The cell suspension was then mixed in 2:1 ratio with Matrigel and 1 ml was added per well. After 30 min incubation at 37 °C Waymouth media was added on top. Cells were treated either with 300 μM *N*-acetyl cysteine (Sigma) or vehicle (water) and media were changed every day. The size of ductal structures was quantified at day 3 using Fiji ImageJ v1.54.

### Western blot

Cells were lysed with RIPA buffer (Millipore) supplemented with phosphatase inhibitor cocktail (Thermo Fisher Scientific) and complete protease inhibitors (Roche) after pelleting. Then, proteins were separated using precast 4–12% Bis–Tris gels (Invitrogen) and transferred to nitrocellulose by dry transfer (iBlot, Thermo Fisher Scientific). Transfer and loading were assessed by rouge ponceau. Membranes were probed with the following primary antibodies: ALDH1L2 (HPA039481) from Atlas Antibodies, vinculin (sc-73614) from Santa Cruz Biotechnology, amylase (D55H10, 3796) from Cell Signaling, CK19 from Abcam (AB52625) and actin (4970) from Cell Signaling. For Extended Fig. 2h, Revert 700 Total Protein stain (Licor) was used as indicated by the manufacturer. All antibodies were used at a dilution of 1:1,000 and were developed with ECL chemiluminescence kits (Pierce) after incubation with the appropriate species-specific horseradish peroxidase-conjugated antibodies. All antibodies were verified and confirmed for species as per the manufacturers’ disclosures. Where appropriate filters were stripped and reprobed with different antibodies.

For Fig. 2h, serum was collected from mice undergoing acute pancreatitis, 1 h after the last injection on day 2 and diluted 1/150. Circulating amylase was measured with the Amylase Assay Kit from Abcam (ab102523) according to the manufacturer’s instructions. The assay was read on a spectramax Plus 384.

### NMR

Nuclear magnetic resonance (NMR) was used to measure formate and valine in cell culture supernatants, plasma, normal interstitial fluid and TIF. For formate quantification in cell culture supernatant as shown in Fig. 4c and Extended Data Fig. 4b, 160 μl of supernatant was collected. As shown in Fig. 4a,b,f,g and Extended Data Fig. 4a,f,g, plasma and TIF were collected and 20 μl were diluted up to 10× in PBS. A solution of 10 mM sodium 2,2-dimethyl-2-silapentane-5-sulfonate (DSS; Sigma) diluted in D_2_O was added to the samples to obtain a final concentration of 1 mM DSS. NMR spectra were acquired at 25 °C with a Bruker Avance III HD instrument with a nominal ^1^H frequency of 700 MHz using 3 mm tubes in a 5 mm CPTCI cryoprobe. For ^1^H 1D profiling spectra, the Bruker pulse program zgesgppe for excitation sculpting with pure echo^58^ was used with a 20 ppm sweep width, 1 s relaxation delay and 4 s acquisition time. Typically, 128 or 256 (for plasma and TIF) transients were acquired. Data were processed and analysed using the Chenomx NMR Suite (Chenomx). Free induction decays were zero filled, apodized with exponential multiplication (line-broadening factor LB of 1 Hz), Fourier transformed and the resulting spectra were then phase corrected before baseline correction, all in the Processor component of the Chenomx software. Formate quantitation was performed based on the chemical shift reference (DSS) assumed to be at 1 mM concentration and with line width adjusted to obtain a good fit to the Chenomx library spectra for multiple metabolites in the spectrum.

### LC–MS

For liquid chromatography–mass spectrometry (LC–MS) detection of plasma formate in Fig. 4h–l and Extended Data Fig. 4c,h–l, 10 μl of plasma was extracted with 390 μl of ice-cold extraction buffer (methanol/acetonitrile/water in the ratio, 50:30:20, v/v/v) containing ^13^C,D-formate (CDLM-6203) from Cambridge Isotope Laboratories, vortexed and spun down at 15,000*g* for 12 min at 4 °C. The supernatant was then dried in a SpeedVac. Dry samples were resuspended in 100 μl of a cold derivatization mix (1/1/3) containing 1,2-^13^C_2_-acetate (CLM440-1) from Cambridge Isotope Laboratories and composed of EDC (11.5 mg ml^−1^ in MeOH; Sigma, E1769) 3-nitrophenylhydrazine (23.5 mg ml^−1^ in MeOH; Sigma, N21804) and Pyridine (40 μl in 1 ml of MeOH), which was incubated for 1 h at 4 °C with three cycles of 10 min sonication. Samples were then centrifuged at 15,000*g* for 12 min and 20 μl of the supernatant was added to 200 μl of β-mercaptoethanol (500 mM). Samples were centrifuged again at 15,000*g* for 12 min before being transferred to vials.

Metabolite analysis was performed by injecting 5 µl sample into a Vanquish Flex UHPLC system (Thermo Scientific) coupled to a Q Exactive Plus Orbitrap mass spectrometer (Thermo Scientific). Analytes were separated using a Waters Acquity BEH C18 column (2.1 × 100 mm, 1.7 μm particle size). The temperature of the column was kept at 60 °C and the flow rate was set to 0.2 ml min^−1^.The elution buffers were water for buffer A and methanol for buffer B. Gradient elution started from 10% of buffer B and was programmed as follows: 0–1 min: 10% B; 2–4 min: 15% to 30% B; 4–5 min: 20% to 100%; 5–7 min: 100% B; 7–7.2 min: 100% to 15% B at a flow rate of 0.2 ml min^−1^. The MS experiment was performed using electrospray ionization in negative mode. Source parameters were applied as follows: sheath gas flow rate, 55; aux gas flow rate, 10; sweep gas flow rate, 0; spray voltage, 3.2 kV (−); capillary temperature, 325 °C; S-lense RF level, 50; aux gas heater temperature, 100 °C. The Orbitrap mass analyser was operated at a resolving power of 70,000 (at 200 *m*/*z*) in selective ion monitoring (SIM) mode (mass targeted: *m*/*z* 180.0414 (formate), 196.0638 (1,2-^13^C_2_-acetate)) and normalized gain control target set to 70% with a maximum injection time of 200 ms. All data were acquired in profile mode with the Thermo Xcalibur software (Version 4.2.47).

Data were processed using XCMS, using the centWave algorithm for peak detection. To correct for batch effects, the LOESS algorithm^59^ was applied using a span parameter of 0.6.

For detection of GSH and GSSG by UPLC–MS (Fig. 2c and Extended Data Figs. 2e–g), pancreatic tissues were dried overnight using a freeze dryer and ground the next morning using disposable pestles. Then samples were extracted using a ratio of 100 μl of extraction mix (methanol/acetonitrile/water in the ratio 50:30:20, v/v/v) for 1 mg of dry tissue. Samples were vortexed, sonicated three times (8 min pulse) and centrifuged (16,000*g*, 20 min, 4 °C) and the supernatant was collected. Pellets were re-extracted using the same protocol with a ratio of 150 μl extraction mix for 3 mg of tissue. Supernatants were then pooled, dried and resuspended in 30 μl of extraction mix before the analysis. Metabolite analysis was performed the same instrumentation as above, using an Agilent Infinity Poroshell 120 HILIC (2.1 × 150 mm, 1.7 µm) for chromatographic separation. The following LC parameters were used: injection volume, 5 μl; column oven temperature, 25 °C; autosampler temperature, 4 °C. Chromatographic separation was achieved using gradient elution at a constant flow rate of 300 μl min^−1^ over a total run time of 19 min. An initial mobile phase of 80% solvent B was held for 2 min, decreased to 5% over 10 min, held 5% for 2 min and finally re-equilibrated to 80% B for 5 min. Solvent A was 0.01% of formic acid and solvent B was acetonitrile. MS was performed in positive ion mode with the following parameters: spray voltage 3.5 kV; probe temperature 320 °C; sheath and auxiliary gases were 30 and 5 arbitrary units, respectively; full scan range: 80–1,000 *m*/*z* with an AGC target set at 1e^6^ and resolution at 70,000. SIM was performed to detect reduced and oxidized glutathione and their respective isotopologues. The parameters used for SIM were as follows: resolution 70,000, AGC target 3e^6^, maximum IT 200 ms, isolation window 0.4 *m*/*z*, spectra were centroid and collision energies were set individually in high-energy collisional dissociation mode. Reduced and oxidized glutathione standards were prepared and analysed in the same batch. Metabolites were identified by comparison of accurate mass, fragmentation and retention time to authentic chemical standards. Data were processed using TraceFinder 4.1 EFS software (Thermo Scientific).

### Human samples

The collection of samples was overseen by Tissue Solutions. Ethical approval for collection of plasma samples from human participants, under the study title ‘Genomics, proteomics and biomarker research of human diseases using human biospecimens’ was obtained from the Independent Ethical Committee of The State health institution of Nizhny Novgorod Region ‘City Clinical Hospital No12’ Nizhny Novgorod, research contract no. NZN12/1 2015 dated 14 October 2015. All patients provided informed consent.

To allow for a relatively equal age and sex distribution across the three groups, we collected an excess number of healthy control and symptomatic controls. Once the *n* = 100 pancreatic cancer samples were collected we selected *n* = 100 samples each from the two control populations, which represented an overall match to age and sex distribution in the pancreatic cancer group.

Pancreatic cancer group inclusion criteria: PDAC at time of diagnosis or pancreatic acinar cell adenocarcinoma at time of diagnosis. Exclusion criteria: has received treatment (radiotherapy, chemotherapy or surgery) for pancreatic cancer or any other cancer, previous history of diagnosed cancer/malignant disease of any type, neuroendocrine pancreas tumours or non-epithelial pancreas tumours.

Symptomatic control group inclusion criteria: chronic pancreatitis at time of diagnosis, acute pancreatitis at time of diagnosis, benign pancreatic pseudo cyst at time of diagnosis, biliary obstruction due to non-malignant disease at time of diagnosis, acute non-malignant cholangitis at time of diagnosis or chronic non-malignant cholangitis at time of diagnosis. Exclusion criteria: history or recent diagnosis of any form of cancer/malignant disease, has received any treatment for pancreas-related disease or family history of pancreas cancer.

Healthy control group inclusion criteria: healthy and within age range of diagnostic population (estimated to be 35–75 years of age). Exclusion criteria: history or recent diagnosis of any form of cancer or malignant disease, diabetes (type I or II), cardiovascular diseases neurodegenerative diseases, any disease/condition of the pancreas or family history of pancreas cancer.

Blood samples were collected in 6 ml lithium–heparin tubes (Greiner Bio-One). Samples were gently mixed, kept at 4 °C and processed within 1 h after collection. Blood was centrifuged for 10 min at 1,500*g*, 4 °C in a horizontal rotor centrifuge to remove erythrocytes. Plasma was then transferred to 15 ml plastic tubes, taking care not to mix blood cells. Tubes with plasma were centrifuged at 2.500*g*, 4 °C in a horizontal rotor centrifuge to remove platelets. Finally, 1 ml aliquots of the plasma were transferred to Eppendorf tubes and stored at −80°C.

### Statistical analysis

All statistical analyses were performed using GraphPad Prism 10 software. An unpaired Student’s *t*-test was performed to compare two groups to each other. If the variance, determined by the *F* test, between the two groups was unequal, a Welch’s correction was applied. For multiple comparisons, a one-way analysis of variance (ANOVA) was used. If the variance between groups, determined by the Brown–Forsythe test, was unequal, a Brown–Forsythe and Welch correction was applied. A *P* value below 0.05 was considered statistically significant. Significance is indicated as follows: *P < 0.05, **P < 0.01, ***P < 0.001 and ****P < 0.0001. The statistical test is mentioned in each figure legend and all test are two sided. All measurements were taken from distinct samples, as noted in the figure legends. In Fig. 4c,g, one and two data points, respectively, were identified as outliers by an outlier test and removed from the dataset.

No statistical methods were used to predetermine sample sizes but our sample sizes are similar to those reported in previous publications. Sample sizes were based on standard protocols in the field. Metabolic data were assigned in a random order before analysis by LC–MS. Mice were randomly assigned to a treatment and mouse experiments were blinded to the person performing metabolomic or immunohistology analysis.

### Immunohistochemistry

Tissues were fixed in 10% neutral buffered formalin for 24 h. The fixed tissues were processed using Sakura Tissue-Tek VIP 6 AI Tissue Processor and paraffin embedded. For H&E staining, 3 μm sections were cut on to microscope slides. The slides were baked at 60 °C for 1 h and stained with Harris Haematoxylin and Eosin using a Sakura Tissue-Tek Prisma auto stainer. For immunohistochemistry, tissues were fixed and embedded as above and 3 μm sections cut on to Plus^+^ Frost positive charged slides (MSS51012BU, Solmedia). The slides were baked at 60 °C for 1 h and then deparaffinized in xylene and rehydrated using a series of graded industrial methylated spirit solutions and distilled water. Heat-mediated antigen retrieval was performed using pH 6 citrate buffer for 20 min in a microwave. Endogenous peroxidase and alkaline phosphatase blocking was performed by incubating the slides in Bloxall (SP-6000-100, Vector) for 10 min at room temperature.

For amylase/CK19 double labelling, protein blocking was performed using a blocking buffer of TBS-T, 10% bovine serum albumin (A3294, Sigma-Aldrich) and 4% milk for 1 h at room temperature. Primary antibody amylase (D55H10, Cell Signaling) was diluted in 1% BSA at a 1:2,000 dilution and incubated for 1 h at room temperature. The slides were then incubated with horse anti-rabbit IgG (BA1100, Vector) for 30 min at room temperature, followed by the VECTASTAIN Elite ABC-HRP kit, peroxidase (PK6100, Vector) for 30 min at room temperature. Slides were then incubated with 3,3-diaminobenzidine (DAB) chromatin (SK-4105, Vector) for 10 min at room temperature. Following this, primary antibody CK19 (Sigma-Aldrich, MABT913) was diluted at a 1:500 dilution and applied to the slides for 1 h at room temperature. The slides were then incubated with the ImmPRESS-AP Goat Anti-Rat IgG Polymer Detection kit, alkaline phosphatase (MP-5404-15) for 30 min at room temperature. The slides were then incubated with the Vector Red Substrate kit, alkaline phosphatase (Vector, SK-5105) for 20 min at room temperature and then counterstained using Harris Haematoxylin and dehydrated, cleared and mounted using Sakura Tissue-Tek Prisma auto stainer. Quantification of ADM in Fig. 2l and Extended Data Fig. 2m was performed on CK19/amylase double staining. The number of double-positive acinar cells and acinar cell losing amylase staining were counted. Total amount of cells in the field of view were counted by the cell detection algorithm in QuPath. For each pancreas, three to six fields of view randomly chosen throughout the slide were quantified in a double blind manner.

For MDA staining, slides were incubated with 2.5% normal horse serum blocking reagent (MP-740) for 30 min at room temperature. Primary antibody MDA (Abcam, ab243066) was diluted at a 1:500 dilution and incubated for 1 h at room temperature, then slides were incubated with ImmPRESS HRP Horse Anti-Rabbit IgG Polymer reagent, peroxidase (Vector, MP-7401) followed by DAB chromatin (SK-4105, Vector) for 10 min at room temperature. Slides were then counterstained using Harris Haematoxylin and dehydrated, cleared and mounted using the Sakura Tissue-Tek Prisma auto stainer. All slides were scanned with a Zeiss AxioScan Z1 and images were generated and quantified with FiJi ImageJ v1.54.

For Picrosirius Red staining, 3 μm sections were cut on to microscope slides. The slides were baked at 60 °C for 1 h then deparaffinized in xylene and rehydrated using a series of graded industrial methylated spirit solutions and distilled water. Slides were incubated in freshly filtered Weigert’s iron haematoxylin solution (Sigma-Aldrich, HT1079) for 10 min. The slides were washed with warm running water for 5 min and then rinsed with distilled water. The slides were incubated in Picrosirius Red solution (Abcam, ab150681) for 1 h and then drained on absorbent paper. The slides were then rinsed with agitation in 0.5% acetic acid, rinsed in 100% ethanol for 2× 1 min, cleared in xylene for 2× 1 min and mounted using Sakura Tissue-Tek glass.

For Fig. 2j, sample embedding, sectioning and staining were conducted by the CRUK-CI Histology Core. ADM area was quantified on H&E sections using Halo Software (Indica Labs).

### RNAscope

For RNAscope, 5 μm sections were cut onto Plus+ Frost positive charged slides (MSS51012BU, Solmedia) and baked at 60 °C for 1 h. Slides were pretreated using the RNAscope 2.5 HD Brown kit pretreatment reagents (ACD, 322300). Following pretreatment, the slides were incubated with the *ALDH1L2*-specific target probe. For mouse samples, the *Aldh1l2* probe (ACD, 447671) was utilized, while for human samples, the corresponding *ALDH1L2* probe (ACD, 1175261-C1) was employed. Slides were then incubated in a series of RNAscope 2.5 HD Brown Kit Detection reagents (ACD, 322300) and counterstained in Harris Haematoxylin, dehydrated, cleared and mounted using Sakura Tissue-Tek Prisma auto stainer. Slides were scanned with a Zeiss AxioScan Z1 at 20× magnification.

### Flow cytometry

For Figs. 1f,g and Extended Data Figs. 1b,c and 3k, pancreata were weighed, then mechanically dissociated, followed by digestion in 5 ml of HBSS containing collagenase I (375 U ml^−1^), DNase I (0.15 mg ml^−1^) and Soybean Trypsin inhibitor (Sigma, 0.05 mg ml^−1^) for 30 min at 37 °C on a shaker (220 rpm), followed by dissociation with a syringe and needle, filtration through a 70-μm strainer to exclude Langerhans islets and red blood cell lysis. Single-cell suspensions from pancreata were used to isolate acinar cells (LiveCD45^−^EpCAM^+^ CD133^lo^ SCC^hi^), ductal cells (LiveCD45^−^EpCAM^+^CD133^+^SSC^lo^) and fibroblasts (LiveCD45^−^EpCAM^−^CD90^+^Podoplanin^+^) by flow cytometry using a FACS Aria instrument. The following antibodies were used in this study with clones, venders and fluorochrome as indicated: CD45 (30-F11, Biolegend, BV510), EpCAM (G8.8, Biolegend, BV711), CD133 (315-2C11, Biolegend, PEDazzle594) and Podoplanin (8.1.1, Biolegend, PE-Cy7). Dead cells were excluded with the fixable viability dye UV455 or eFluor780 (eBioscience). Data were analysed using FlowJo X (Tree Star).

For Fig. 2a, cells were incubated with 5 mM of MitoSox Red mitochondrial superoxide indicator (M36008, Thermo Fisher Scientific) for 10 min at 37 °C. Cells were washed with PBS 1 mM, EDTA 2.5% FBS and filtered into FACS tubes. Samples were run on a FACS Fortessa with data acquired with FACSdiva and analysed with FlowJo (10.7.2).

### RT–qPCR

For Fig. 1f,g and Extended Data Figs. 1b,c and 3k, RNA from single-cell suspensions of acinar, ductal and stromal cells was extracted using Tri-Reagent (Sigma) followed by 1-bromo-3-chloropropane extraction and clean-up on RNeasy columns. For Fig. 4d,e and Extended Data Fig. 4d RNA from total pancreas lysates was prepared. After killing, pancreata were perfused with 500 μl RNA Later (Ambion) and snap frozen in liquid nitrogen. Upon thawing, tissue was directly transferred into CK28 tubes (Precellys) filled with 5 ml Tri-Reagent (Sigma) and homogenized for 1× 30 s at 7,200 rpm using a Precellys homogenizer. RNA extraction from homogenates was performed following the Tri-Reagent protocol, with the addition of an isopropanol washing step before ethanol precipitation. RNA quality was assessed using the Agilent Tapestation. RNA was converted into cDNA using the High-Capacity RNA-to-cDNA kit (Thermo Scientific), followed by qPCR using the Takyon Low Rox Probe Master mix dTTP Blue (Eurogentec) and the following primers/probe predesigned assays (Integrated DNA Technologies): *Hprt* (Mm.PT.39a.22214828), *Cpa1* (Mm.PT.58.15958828), *Krt19* (Mm.PT.58.7322803), *Pdgfra* (Mm.PT.56a.5639577), *Aldh1l2* (Mm.PT.56a.12885445), *Aldh1l1* (Mm.PT.58.7775479), *Shmt2* (Mm.PT.58.41661954), *Mthfd2* (Mm.PT.58.43172230), *Mthfd1l* (Mm.PT.58.23357428) and *Tigar* (Mm.PT.56a.16927616).

### Reporting summary

Further information on research design is available in the Nature Portfolio Reporting Summary linked to this article.

## Supplementary information

Reporting Summary

## Source data

Source Data Fig. 1 Statistical source data of Fig. 1.

Source Data Fig. 1 Gel source data of Fig. 1.

Source Data Fig. 2 Statistical source data of Fig. 2.

Source Data Fig. 3 Statistical source data of Fig. 3.

Source Data Fig. 3 Gel source data of Fig. 3.

Source Data Fig. 4 Statistical source data of Fig. 4.

Source Data Extended Data Fig. 1 Statistical source data of Extended Data Fig. 1.

Source Data Extended Data Fig. 2 Statistical source data of Extended Data Fig. 2.

Source Data Extended Data Fig. 2 Gel source data of Extended Data Fig. 2.

Source Data Extended Data Fig. 3 Statistical source data Extended Fig. 3

Source Data Extended Data Fig. 3 Gel source data Extended Fig. 3

Source Data Extended Data Fig. 4 Statistical source data Extended Fig. 4

## Figures and Tables

**Extended Data Table 1:**
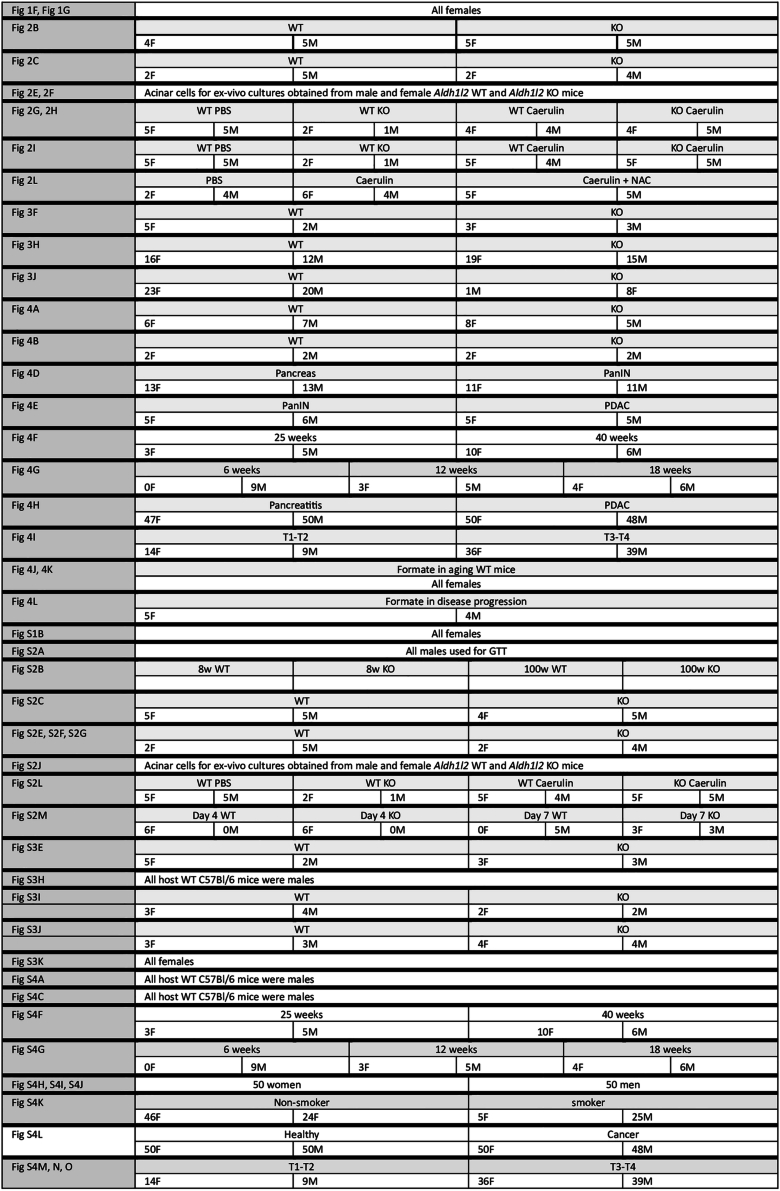
Distribution of male and female mice for in vivo experiments

## Data Availability

For Fig. 1a and Extended Data Fig. 1a, data were extracted from the consensus dataset of the human protein atlas. For Extended Data Fig. 1d–f, data were retrieved and extracted from the NCBI gene expression omnibus repository, accession number GSE240106. For Extended Data Fig. 1c, data were retrieved and extracted from the NCBI gene expression omnibus repository, accession number GSE179248. For Fig. 3b,c and Extended Data Fig. 3a, data were obtained from http://gepia.cancer-pku.cn/. Source data are provided with this paper.
