# Supplementary material for: ALDH1L2 regulates reactive oxygen species and acinar-to-ductal metaplasia in the pancreas
Source: Nat Metab. 2026 Apr 1;8(4):810–23. doi: 10.1038/s42255-026-01456-5 (PMC13120998; doi:10.1038/s42255-026-01456-5)
Supplement: Supplementary file 1 — Reporting Summary [file 42255_2026_1456_MOESM1_ESM.pdf]

nature portfolio

Corresponding author(s): Dr Karen Vousden
Dr Marc Hennequart

Last updated by author(s): Nov 10, 2024

Reporting Summary

Nature Portfolio wishes to improve the reproducibility of the work that we publish. This form provides structure for consistency and transparency in reporting. For further information on Nature Portfolio policies, see our Editorial Policies and the Editorial Policy Checklist.

Statistics

For all statistical analyses, confirm that the following items are present in the figure legend, table legend, main text, or Methods section.

n/a	Confirmed	
☐	☒	The exact sample size (*n*) for each experimental group/condition, given as a discrete number and unit of measurement
☐	☒	A statement on whether measurements were taken from distinct samples or whether the same sample was measured repeatedly
☐	☒	The statistical test(s) used AND whether they are one- or two-sided<br>*Only common tests should be described solely by name; describe more complex techniques in the Methods section.*
☐	☒	A description of all covariates tested
☐	☒	A description of any assumptions or corrections, such as tests of normality and adjustment for multiple comparisons
☐	☒	A full description of the statistical parameters including central tendency (e.g. means) or other basic estimates (e.g. regression coefficient) AND variation (e.g. standard deviation) or associated estimates of uncertainty (e.g. confidence intervals)
☐	☒	For null hypothesis testing, the test statistic (e.g. *F*, *t*, *r*) with confidence intervals, effect sizes, degrees of freedom and *P* value noted<br>*Give P values as exact values whenever suitable.*
☒	☐	For Bayesian analysis, information on the choice of priors and Markov chain Monte Carlo settings
☒	☐	For hierarchical and complex designs, identification of the appropriate level for tests and full reporting of outcomes
☒	☐	Estimates of effect sizes (e.g. Cohen's *d*, Pearson's *r*), indicating how they were calculated

Our web collection on statistics for biologists contains articles on many of the points above.

Software and code

Policy information about availability of computer code

Data collection	For NMR: For 1H 1D profiling spectra the Bruker pulse program zgespe for excitation sculpting with pure echo (Adams R.W, et al. Chem Commun (Camb), 2013. 49(4): p.358-360) was used with 20 ppm sweep width, 1 s relaxation delay and 4 s acquisition time.<br>RT-qPCR data were collected on a QuantStudio 3 system from applied biosystems.
Data analysis	For NMR: Data were processed and analysed using Chenomx NMR Suite (Chenomx, Edmonton, Canada).<br>For LC-MS: Data were analysed with TraceFinder and Xcalibur from Thermofisher, to correct for batch effects, the LOESS algorithm54 was applied using a span parameter of 0.6.<br>All slides were scanned with Zeiss AxioScan Z1 and images generated and quantified with positive cell detection algorithm on QuPath. ADM area was quantified on H&E sections using Halo Software (Indica Labs).<br>All statistical analyses and graphs were performed using GraphPad Prism 10 software

For manuscripts utilizing custom algorithms or software that are central to the research but not yet described in published literature, software must be made available to editors and reviewers. We strongly encourage code deposition in a community repository (e.g. GitHub). See the Nature Portfolio guidelines for submitting code & software for further information.

Data

Policy information about availability of data

All manuscripts must include a data availability statement. This statement should provide the following information, where applicable:

- Accession codes, unique identifiers, or web links for publicly available datasets
- A description of any restrictions on data availability
- For clinical datasets or third party data, please ensure that the statement adheres to our policy

All the data (including source data) supporting the findings of this study are available within the article, the supplementary information files and the source data file.

Research involving human participants, their data, or biological material

Policy information about studies with human participants or human data. See also policy information about sex, gender (identity/presentation), and sexual orientation and race, ethnicity and racism.

Reporting on sex and gender	All samples were prospectively collected for this study. In order to allow for a relatively equal age and sex distribution across the three groups we prospectively collected an excess number of healthy control and symptomatic controls. Sex was included as a covariate and plasma levels of formate were compared in between male and female in order to assess if sex had an influence on this parameter.
Reporting on race, ethnicity, or other socially relevant groupings	All patients that provided samples were ethnically Caucasian (white) from Russian heritage.
Population characteristics	Pancreatic Cancer Group: Inclusion Criteria: Pancreatic ductal adenocarcinoma at time of diagnosis, pancreatic acinar cell adenocarcinoma at time of diagnosis. Exclusion Criteria: Has received treatment (radiotherapy, chemotherapy, surgery) for pancreatic cancer or any other cancer, previous history of diagnosed cancer / malignant disease of any type, neuroendocrine pancreas tumours, non-epithelial pancreas tumours.<br><br>Symptomatic Control Group: Inclusion Criteria: Chronic pancreatitis at time of diagnosis, acute pancreatitis at time of diagnosis, benign pancreatic pseudo cyst at time of diagnosis, biliary obstruction due to non-malignant disease at time of diagnosis, acute non-malignant cholangitis at time of diagnosis, chronic non-malignant cholangitis at time of diagnosis. Exclusion Criteria: History or recent diagnosis of any form of cancer / malignant disease, has received any treatment for pancreas related disease, family history of pancreas cancer.<br><br>Healthy Control Group: Inclusion criteria: Healthy, within age range of diagnostic population (estimated to be 35-75 years of age. Exclusion criteria: History or recent diagnosis of any form of cancer or malignant disease, diabetes (type I or II), cardiovascular diseases neurodegenerative diseases, any disease/condition of the pancreas, family history of pancreas cancer.
Recruitment	Collection of samples was overseen by Tissue Solutions (Glasgow, UK). All samples were prospectively collected for this study. In order to allow for a relatively equal age and sex distribution across the three groups we prospectively collected an excess number of healthy control and symptomatic controls. Once the n=100 Pancreatic Cancer samples were collected we selected n=100 samples each from the two control populations which represented an overall match to age and sex distribution in the Pancreatic Cancer group.
Ethics oversight	Ethical approval for collection of plasma samples from human subjects, under the study tile 'Genomics, Proteomics and Biomarker Research of Human Diseases Using Human Biospecimens' was obtained from the Independent Ethical Committee of The State health institution of Nizhny Novgorod Region "City Clinical Hospital No12" Nizhny Novgorod, research contract # NZN12/1 2015 dated 10/14/2015. All subjects provided informed consent.

Note that full information on the approval of the study protocol must also be provided in the manuscript.

Field-specific reporting

Please select the one below that is the best fit for your research. If you are not sure, read the appropriate sections before making your selection.

☒ Life sciences ☐ Behavioural & social sciences ☐ Ecological, evolutionary & environmental sciences

For a reference copy of the document with all sections, see nature.com/documents/nr-reporting-summary-flat.pdf

Life sciences study design

All studies must disclose on these points even when the disclosure is negative.

Sample size	Experiments were performed using sample sizes based on standard protocols in the field. No statistical test was performed to predetermine sample size.
Data exclusions	All measurements were taken from distinct samples, as noted in the Figure legends. In figure 4C and 4G, one and two data points, respectively, were identified as outliers by an outlier test and removed from the data set.
Replication	Information provided in Figure legends
Randomization	All metabolic data are assigned a random order before being injected through the LC-MS column.<br>For mouse experiments , WT and KO mice are randomly assigned in cages of maximum 5 individuals
Blinding	Mouse experiments were double blinded for the analysis of pancreatitis and pancreatic cancer severity and metabolic data analysis.<br>Human metabolomics were also double blinded.

Reporting for specific materials, systems and methods

We require information from authors about some types of materials, experimental systems and methods used in many studies. Here, indicate whether each material, system or method listed is relevant to your study. If you are not sure if a list item applies to your research, read the appropriate section before selecting a response.

Materials & experimental systems

n/a	Involved in the study	
☐	☒	Antibodies
☐	☒	Eukaryotic cell lines
☒	☐	Palaeontology and archaeology
☐	☒	Animals and other organisms
☒	☐	Clinical data
☒	☐	Dual use research of concern
☒	☐	Plants

Methods

n/a	Involved in the study	
☒	☐	ChIP-seq
☐	☒	Flow cytometry
☒	☐	MRI-based neuroimaging

Antibodies

Antibodies used	Mouse Monoclonal anti-human Vinculin; clone 7F9 ; sc-73614; from Santa Cruz Biotechnology;<br>Rabbit monoclonal anti-human/mouse amylase; clone D55H10; #3796; from Cell Signaling,<br>Rabbit monoclonal anti-human/mouse cytokeratin 19; clone ; EP1580Y ;AB52625; from Abcam<br>Rabbit monoclonal anti-human/mouse beta-Actin; clone 13E5# 4970; from Cell Signaling.<br>Mouse monoclonal anti-Malondialdehyde , clone 11E3, ab243066; from abcam<br>Rabbit Polyclonal anti-human ALDH1L2, HPA039481, form Atlas antibiodies<br>Rat monoclonal anti-human/mouse CK19, clone TROMA-3, MABT913, from Sigma-Aldrich.
Validation	ALDH1L2: https://www.atlasantibodies.com/products/primary-antibodies/triple-a-polyclonals/anti-aldh1l2-antibody-hpa039481/<br>Supplementary validation was performed for anti-ALDH1L2. Antibody was tested on human breast cancer cell lines over-expressing ALDH1L2 or Knock-out for ALDH1L2. Antibody was also validated on WT and KO mouse tissue.<br>Vinculin: https://www.scbt.com/p/vinculin-antibody-7f9<br>CK19: https://www.abcam.com/en-us/products/primary-antibodies/cytokeratin-19-antibody-ep1580y-cytoskeleton-marker-ab52625<br>Amylase: https://www.cellsignal.com/products/primary-antibodies/a-amylase-d55h10-xp-rabbit-mab<br>Actin: https://www.cellsignal.com/products/primary-antibodies/b-actin-13e5-rabbit-mab/4970<br>MDA: https://www.abcam.com/en-us/products/primary-antibodies/malondialdehyde-antibody-11e3-ab243066<br>CK19: https://www.sigmaaldrich.com/GB/en/product/mm/mabt913

Eukaryotic cell lines

Policy information about cell lines and Sex and Gender in Research

Cell line source(s)	All cell lines were provided by the Cell Services depository for the Francis Crick Institute

Authentication	All cell lines were authenticared using STR profiling and species identificationes.
Mycoplasma contamination	All cell lines were negative for mycoplasma upon thawing.
Commonly misidentified lines (See ICLAC register)	*Name any commonly misidentified cell lines used in the study and provide a rationale for their use.*

Animals and other research organisms

Policy information about studies involving animals; ARRIVE guidelines recommended for reporting animal research, and Sex and Gender in Research

Laboratory animals	C57BL/6 were obtained from the in-house breeding facilities (Francis Crick Institute, CRUK Beatson Institute and CRUK Cambridge Institute)<br>For Fig 4C, 4D and Extended Data Figs 4A, Ptf1aCre/+ Kras LSL-G12D mice were provided by Prof. Brindle (CRUK-CI)<br>Tigar KO, Tigar KO pancreatic cancer models and Aldh1l2 KO mice obtained as previously described(ref 27 and 37)<br>For Fig 3, 4, Extended Data Figs 3 and 4, unless indicated otherwise, Trp53+/LSL-R172H, Kras+/LSL-G12D, Trp53+/fl, p48Cre strains were crossed together to obtain KFC (p48Cre; Kras+/LSL-G12D;Trp53+/fl) and KPC (p48Cre, Kras+/LSL-G12D, Trp53+/LSL-R172H) mice. For Aldh1l2 knock-outs, Aldh1l2 KO strain was used to breed into the KFC to obtain KPC and KFC mice bearing Aldh1l2 deletion in C57Bl/6 background<br>For Fig 3H, the acinar cancer model, B6.Cg-Tg(Ela1-TAg*)289Mjt/J was obtained from the Jackson laboratory (#008247) and crossed with the Aldh1l2 KO strain
Wild animals	This study did not involve wild animals.
Reporting on sex	All mouse experiments had a balanced number of male and female animals. Influence of sex on the experimental question was not addressed for this study.
Field-collected samples	This study did not involve samples collected from the field.
Ethics oversight	All experiments were conducted in compliance with the UK Home Office-approved project licences and personal licences (Animals Scientific Procedures Act 1986) and within institutional welfare guidelines of the Francis Crick Institute (reviewed and approved by the Francis Crick Animal Welfare Ethical Review Body) , the CRUK Beatson Institute (reviewed and approved by the University of Glasgow and UK Home Office) and the CRUK Cambridge Institute (with approval from CRUK-CI Animal Welfare Ethical Review Body).

Note that full information on the approval of the study protocol must also be provided in the manuscript.

Plants

Seed stocks	*Report on the source of all seed stocks or other plant material used. If applicable, state the seed stock centre and catalogue number. If plant specimens were collected from the field, describe the collection location, date and sampling procedures.*
Novel plant genotypes	*Describe the methods by which all novel plant genotypes were produced. This includes those generated by transgenic approaches, gene editing, chemical/radiation-based mutagenesis and hybridization. For transgenic lines, describe the transformation method, the number of independent lines analyzed and the generation upon which experiments were performed. For gene-edited lines, describe the editor used, the endogenous sequence targeted for editing, the targeting guide RNA sequence (if applicable) and how the editor was applied.*
Authentication	*Describe any authentication procedures for each seed stock used or novel genotype generated. Describe any experiments used to assess the effect of a mutation and, where applicable, how potential secondary effects (e.g. second site T-DNA insertions, mosiacism, off-target gene editing) were examined.*

Flow Cytometry

Plots

Confirm that:

- [ ] The axis labels state the marker and fluorochrome used (e.g. CD4-FITC).
- [ ] The axis scales are clearly visible. Include numbers along axes only for bottom left plot of group (a 'group' is an analysis of identical markers).
- [ ] All plots are contour plots with outliers or pseudocolor plots.
- [ ] A numerical value for number of cells or percentage (with statistics) is provided.

Methodology

Sample preparation	For Figure 1F, 1G, Extended Data Figs 1B, 1C, 3J, pancreata were weighed, then mechanically dissociated, followed by digest in 5 ml of HBSS containing collagenase I (375 U/ml), DNase I (0.15 mg/ml) and Soybean Trypsin inhibitor (Sigma, 0.05 mg/ml) for 30 minutes at 37°C on a shaker (220 rpm), followed by dissociation with a syringe and needle, filtration through a 70 µm strainer to exclude Langerhans islets, and red blood cell lysis.

Instrument	BD FACS Aria.
Software	BD Diva
Cell population abundance	None
Gating strategy	Sorting of exocrine pancreas cells was generated in Stockis et al. 2024 (https://doi.org:10.1101/2024.02.15.580302 ; reference 30), and cDNA library was prepared on these samples. Gating strategy and figure of the gating strategy can be found in reference 30.

☐ Tick this box to confirm that a figure exemplifying the gating strategy is provided in the Supplementary Information.
