# Supplementary figures and images for: ALDH1L2 regulates reactive oxygen species and acinar-to-ductal metaplasia in the pancreas
Source: Nat Metab. 2026 Apr 1;8(4):810–23. doi: 10.1038/s42255-026-01456-5 (PMC13120998; doi:10.1038/s42255-026-01456-5)

Figure 1B

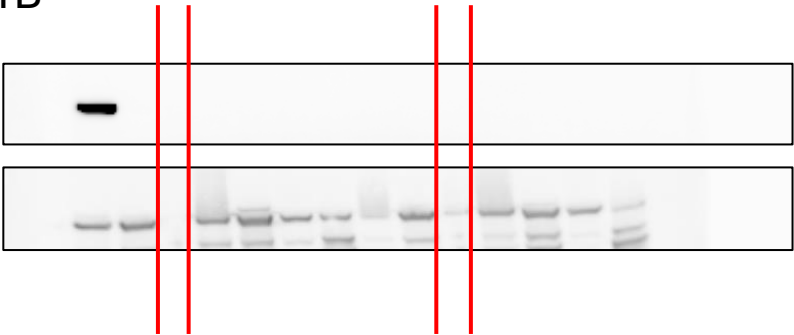

Figure 1H

ALDH1L2

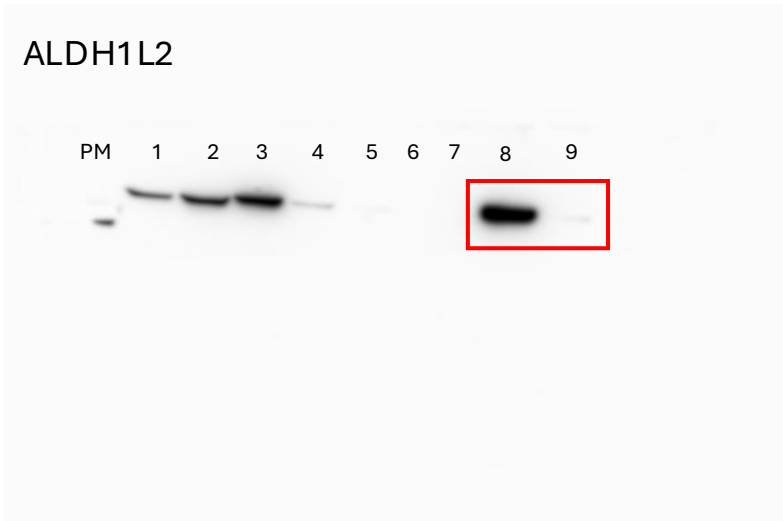

CK19

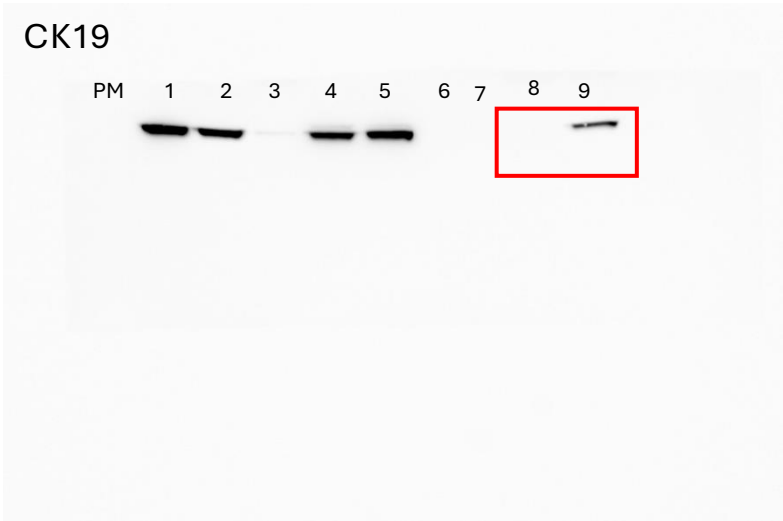

Amylase

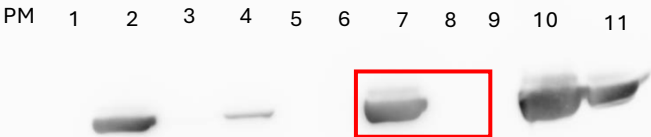

Vinculin

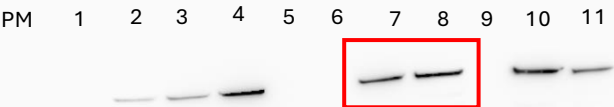

Supplement: Supplementary file 3 — Gel source data of Fig. 1. [file 42255_2026_1456_MOESM3_ESM.pdf]

Figure 3D

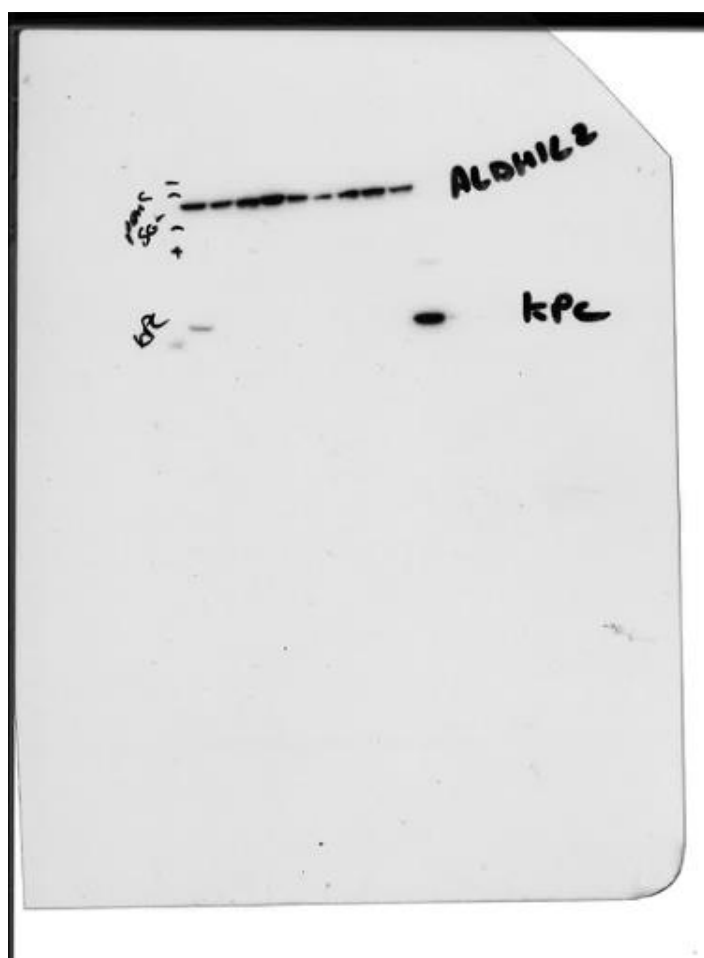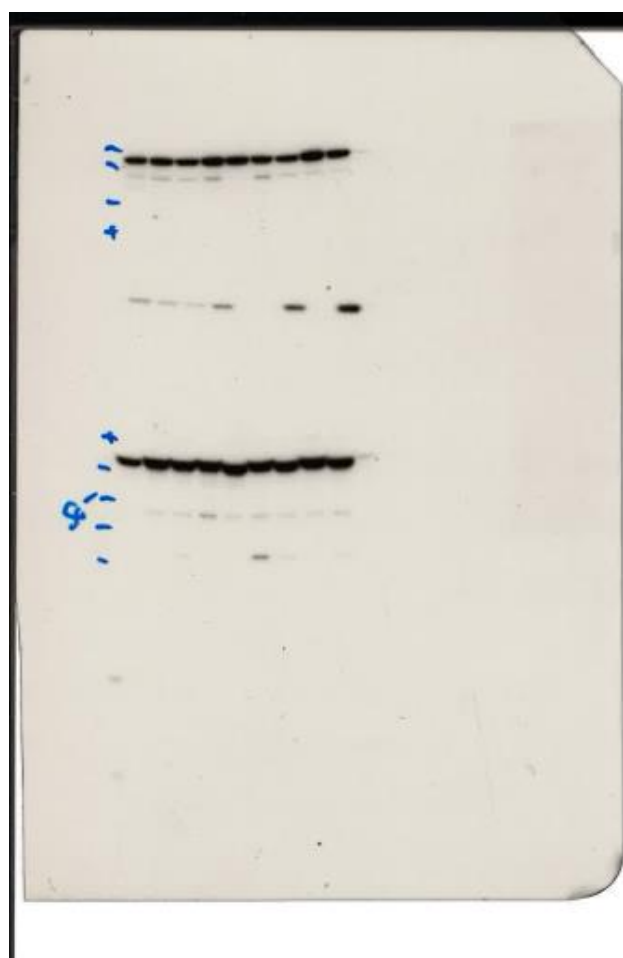

Supplement: Supplementary file 6 — Gel source data of Fig. 3. [file 42255_2026_1456_MOESM6_ESM.pdf]

Figure Ext 2H

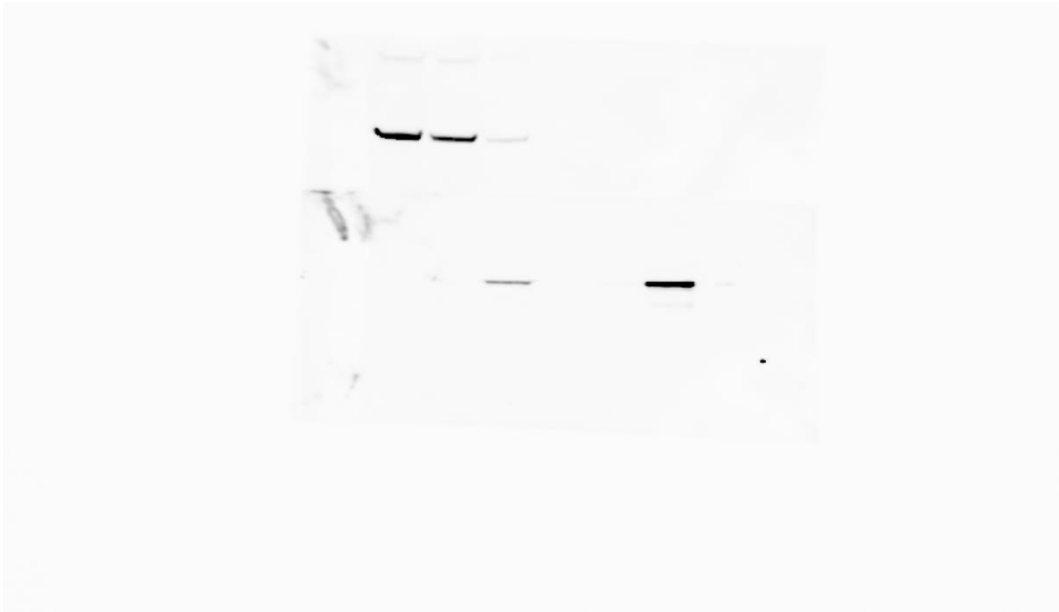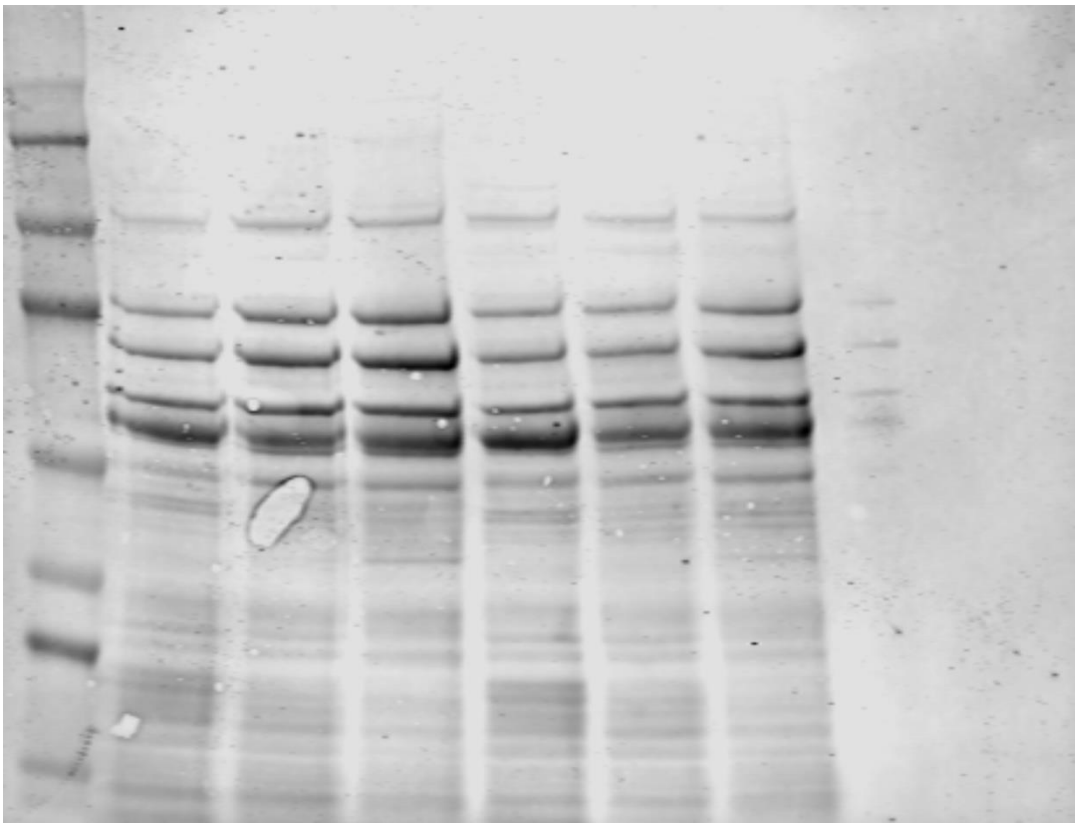

Supplement: Supplementary file 10 — Gel source data of Extended Data Fig. 2. [file 42255_2026_1456_MOESM10_ESM.pdf]

Figure Ext 3B

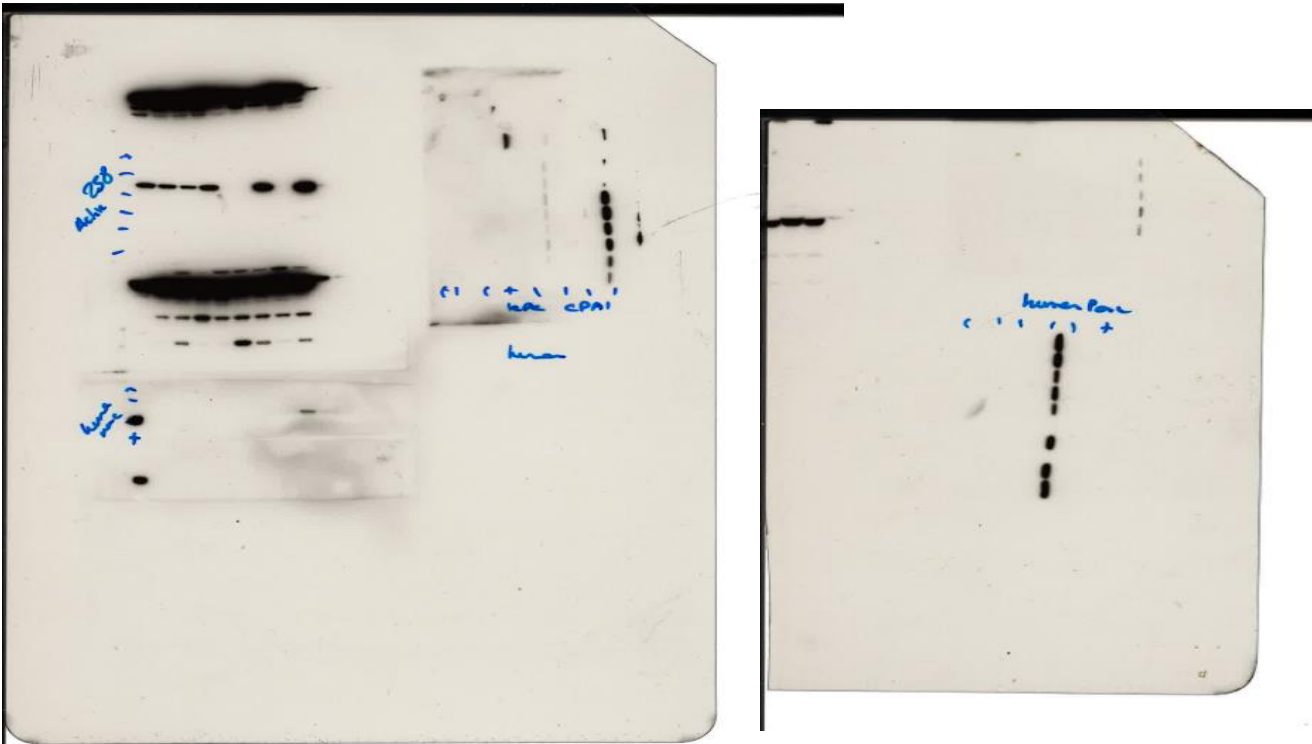

Figure Ext 3G

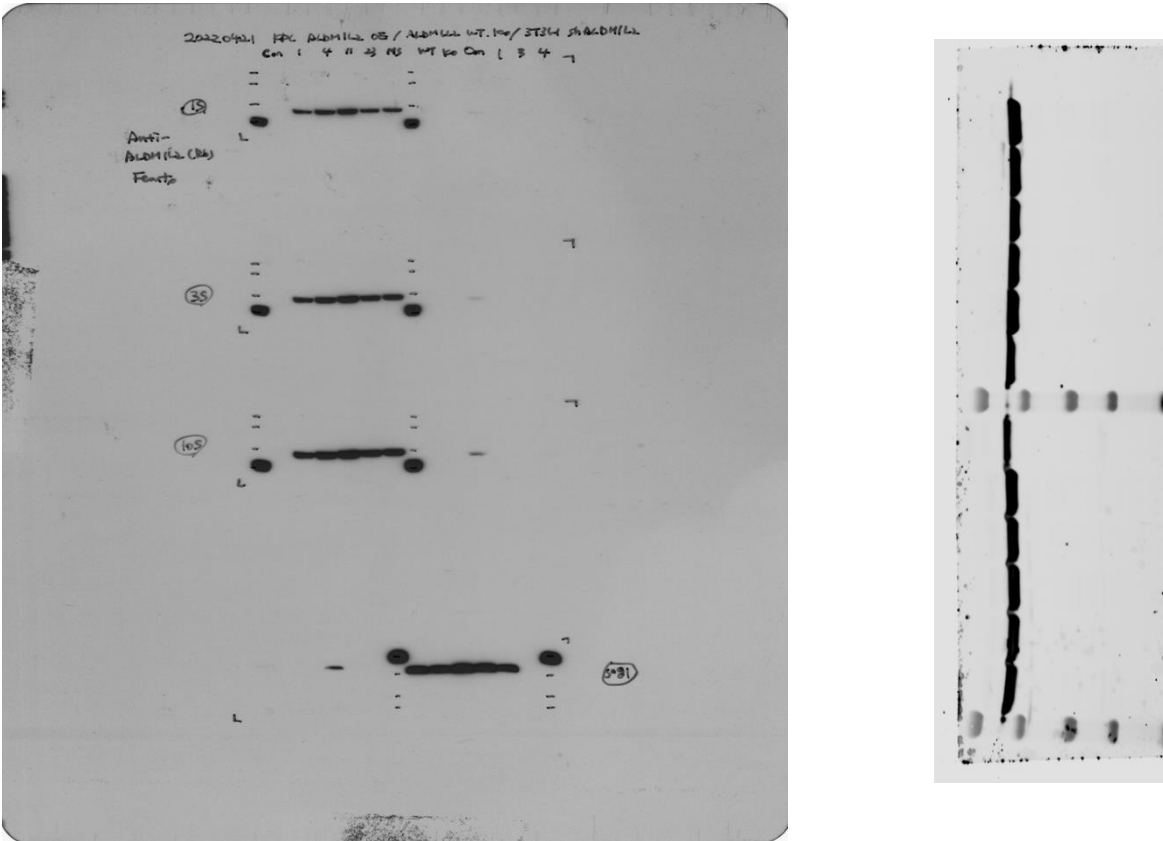

Supplement: Supplementary file 12 — Gel source data Extended Fig. 3 [file 42255_2026_1456_MOESM12_ESM.pdf]
